# Comparative efficacy and safety of daridorexant, lemborexant, and suvorexant for insomnia: a systematic review and network meta-analysis

**DOI:** 10.1038/s41398-025-03439-8

**Published:** 2025-06-24

**Authors:** Taro Kishi, Toshikazu Ikuta, Leslie Citrome, Kenji Sakuma, Masakazu Hatano, Shun Hamanaka, Yasufumi Nishii, Nakao Iwata

**Affiliations:** 1 Department of Psychiatry, Fujita Health University School of Medicine, Toyoake, Aichi Japan; 2 Department of Communication Sciences and Disorders, School of Applied Sciences, University of Mississippi, University, MS USA; 3 Department of Psychiatry and Behavioral Siences, New York Medical College, Valhalla, NY USA; 4 Department of Pharmacotherapeutics and Informatics, Fujita Health University School of Medicine, Toyoake, Aichi Japan

**Keywords:** Psychiatric disorders, Addiction

## Abstract

**Background:**

In order to appraise the risk-benefit balance of the three available dual orexin receptor antagonists (DORAs; daridorexant, lemborexant, and suvorexant) for the management of adults with insomnia, we conducted a systematic review and random-effects model network meta-analysis.

**Methods:**

Included were all published double-blind, randomized, placebo-controlled trials of these agents. Outcomes included subjective time to sleep onset at month 1 (sTSO, primary), subjective total sleep time at month 1 (sTST, co-primary), subjective wake after sleep onset at month 1, Insomnia Severity Index scores at month 1, all-cause discontinuation, discontinuation due to adverse events, and the incidence of individual adverse events such as somnolence, dizziness, falls, headache, nasopharyngitis, and upper respiratory tract infection.

**Results:**

This meta-analysis included eight trials (5198 adults, average age = 56.33 years, 67.84% female). The treatment arms included daridorexant 25 mg/day (DAR25), daridorexant 50 mg/day (DAR50), lemborexant 5 mg/day (LEM5), lemborexant 10 mg/day (LEM10), suvorexant 20 mg/day (15 mg/day for people ≥65years, SUV20/15), and placebo. All active-treatments outperformed placebo in terms of all efficacy outcomes. The standardized mean difference (95% CI) in primary outcomes ranged from; sTSO: −0.430 (−0.568, −0.292) for LEM10 to −0.164 (−0.296, −0.031) for SUV20/15 and sTST: −0.475 (−0.593, −0.357) for DRA50 to −0.206 ( −0.330, −0.082) for LEM5. An additional sensitivity analysis suggested that DRA25, LEM10, and SUV20/15 were associated with a higher incidence of somnolence compared to a placebo.

**Conclusions:**

Considering that there is no evidence that DORAs are associated with physiological tolerance, withdrawal symptoms, or rebound insomnia when abruptly discontinued, and that sleep architecture is not adversely affected, the DORAs appear to be a favorable choice in managing insomnia disorder in adults.

## Introduction

Insomnia affects approximately one-third of the adult population worldwide [1, 2]. Insomnia is frequently associated with other somatic diseases (such as cardiometabolic diseases) and psychiatric disorders (such as depression) [2–4], resulting in significant health and societal economic costs [5]. Therefore, treatment for insomnia should be provided regardless of whether or not any comorbid illnesses may have contributed to insomnia [2]. In practice, pharmacotherapy is frequently used and may be preferred by the patient [6]. According to the National Health Interview Survey, 8.4% of adults in the United States used sleep medication every day or most days in the previous 30 days to treat insomnia in 2020 [7]. The appropriate drug selection is based on the patient’s symptoms, which may include difficulty initiating and maintaining sleep, as well as early morning awakening with an inability to return to sleep. It is critical to select an insomnia medication that is both effective and safe [6].

The major categories of drugs authorized by the United States Food and Drug Administration (FDA) for the treatment of insomnia include benzodiazepines (BZDs), non‐BZDs (“Z‐drugs”), melatonin receptor agonists, and most recently, dual orexin receptor antagonists (DORAs) [8]. Gamma‐aminobutylic acid type A (GABA_A_) receptors are the targets of BZDs and Z‐drugs, and they modulate inhibitory neurotransmission in the brain. Therefore, while these drugs may be anxiolytic, anticonvulsant, and muscle relaxant, they may cause cognitive impairment such as learning, attention, memory, and occurrence of injurious falls, road traffic, and other accidents [6]. Because placebo-controlled randomized studies of sudies of BZDs have generally been limited to short periods of time (≤4 weeks) [9], BZDs have not been proven to be safe for more than 2–4 weeks. Moreover, approximately half of patients who use BZDs for more than 1 month develop dependence [10–12]. The FDA mandated product label warnings that BZDs and Z‐drugs could lead to physical dependence and induce sleep‐related complex behavior while not fully awake [13]. The Japanese Pharmaceuticals and Medical Devices Agency has issued an alert regarding the risks of administering BZDs and Z‐drugs for prolonged periods administration to avoid these risks [14]. Furthermore, BZDs and Z-drugs are on the Beers List as inappropriate for use in older adults [15].

Although melatonin receptor agonists and DORAs have not been linked to physiological dependence [6], a recent network meta-analysis found that ramelteon, a melatonin receptor agonist, did not improve sleep quality in people with insomnia [9]. However, our meta-analysis and other pooled analysis revealed that lemborexant (LEM) and suvorexant (SUV), both classified as DORAs, have a desirable risk–benefit balance for treating insomnia in adults [16, 17]. Daridorexant (DAR) which was more recently commercialized, was also shown to have a favorable risk-benefit balance for indivuduals with insomnia by a post hoc analysis of a 3-month pivotal Phase 3 study of DAR [18]. To determine whether there are differences in the efficacy, acceptability, tolerability, and safety profiles of DAR, LEM, and SUV, we conducted a systematic review and random-effects model network meta-analysis that included double-blind, randomized, placebo-controlled trials of DORAs. The recommended or approved doses for DORAs varies by country [13, 14, 19]. Therefore, this network meta-analysis included the following treatment arms: DAR 25 mg/day (DAR25), DAR 50 mg/day (DAR50), LEM5, LEM10, SUV 20 mg/day (15 mg/day for people <65 years, SUV20/15), and placebo.

## Materials and methods

This study followed the Preferred Reporting Items for Systematic Reviews and Meta-Analyses guidelines [20] (Table S1) and Cochrane Handbook for Systematic Reviews of Interventions [21], and was registered on the Open Science Framework (https://osf.io/mce9t). The literature search, data transfer accuracy, and calculations were verified by at least two of the authors.

### Search strategy and inclusion criteria

A systematic literature review was conducted using the “PICO” strategy as follows: the participants were adults with insomnia, the intervention was DAR, LEM, or SUV, the control was a placebo or other active treatments, and the outcomes were efficacy, acceptability, tolerability, and safety, as detailed in the following section. The studies’ inclusion criteria were as follows: (1) double-blind, randomized, placebo-controlled trials lasting at least 4 weeks that included DAR, LEM, or SUV; and (2) studies involving adult patients with insomnia (as defined by any recognized diagnostic criteria). Our study’s exclusion criteria were as follows: (1) studies with pediatric or adolescent patients with insomnia; and (2) studies that included only an unrecommended dose arm in the United States [13], Europe [19], or Japan [14]. We searched the PubMed, the Cochrane Library, and Embase databases for studies published before September 21, 2024, without language restriction. The search terms for PubMed and the Cochrane Library included (daridorexant OR suvorexant OR lemborexant) AND (random*) AND (insomnia). The search terms for Embase included (‘lemborexant’/exp OR lemborexant OR ‘suvorexant’/exp OR suvorexant OR ‘daridorexant’/exp OR daridorexant) AND (‘randomized controlled trial’/exp OR ‘randomized controlled trial’) AND (‘insomnia’/exp OR insomnia). Additionally, reference lists of the included articles and the review articles [22–24] were manually searched for additional relevant published and unpublished research, including conference abstracts. We also searched clinical trial registries (ClinicalTrials.gov [http://clinicaltrials.gov/] and the World Health Organization International Clinical Trials Registry Platform [http://www.who.int/ictrp/search/en/]) to ensure the trials were comprehensive and to minimize the effect of publication bias. Any discrepancies in the selected articles were resolved by consensus of the authors. If multiple papers or academic conference abstracts were reported despite the same research, the literature was screened by confirming the clinical trial registration number and/or reference to past review articles [22–24].

### Outcome measures, data synthesis, and data extraction

The efficacy outcomes of this systematic review and meta-analysis were subjective time to sleep onset (sTSO) at month 1 (primary), subjective total sleep time (sTST) at month 1 (co-primary), subjective wake after sleep onset (sWASO) at month 1, and Insomnia Severity Index (ISI) [25] scores at month 1. Other outcomes included all-cause discontinuation (treatment acceptability), discontinuation due to adverse events (treatment tolerability), and the frequency of individual adverse events such as somnolence, dizziness, falls, headache, nasopharyngitis, and upper respiratory tract infection (treatment safety). For sTST at month 1, the algebraic sign of the numerical scores was reversed because lower scores indicated a greater impairment. We conducted a network meta-analysis for the outcomes which included at least five trials. The extracted data were analyzed using the intention-to-treat or modified intention-to-treat principles. We did not use completer analysis data. If required data were missing from the studies, we searched for the data in published systematic review article [4, 6, 16].

### Meta-analysis methods

The frequentist network meta-analysis employed a random-effects model [26, 27]. The standardized mean difference (SMD) for continuous variables and the odds ratio (OR) for dichotomous variables were calculated, along with 95% confidence intervals (95% CI). The network heterogeneity was assessed with *τ*^2^ statistics. The design-by-treatment test (globally) and the Separating Indirect from Direct Evidence test (locally) were used to statistically evaluate incoherence [28, 29]. The treatments for each outcome were ranked using surface under the curve cumulative ranking probabilities. We determined the sufficiency of the distribution differences to validate the analysis by comparing the distribution of possible effect modifiers across included treatments in the network meta-analysis using the Kruskal–Wallis test (continuous variables) and the Pearson chi-square test or Fisher’s exact test (categorical variables) and by assessing their actual influence on the treatment effect through network meta-regression analyses [30–32]. Potential confounding factors included the proportion of females, mean age, proportion of elderly people (aged ≥65 years), total number of participants, and publication year (Table S2). For acceptability, tolerability, and safety outcomes, where observation periods could not be matched, we performed a sensitivity analysis excluding one long-duration (6 months) study of LEM [33] similar to the recent network meta-analysis that evaluated any outcomes divided acute treatment (<3 months) or and long-term treatment (≥3 months)(i.e., our sensitivity analysis included only 3 month or shorter studies) [9]. We assessed the overall risk of bias for each trial in our systematic review using version 2 of the Cochrane risk of bias tool for randomized trials (https://www.riskofbias.info/). Finally, the results were integrated into the Confidence in Network Meta-Analysis application, which is an adaptation of the Grading of Recommendations Assessment, Development, and Evaluation approach, to assess the credibility of the findings of each of the network meta-analyses [34–36]. Publication bias was evaluated using a funnel plot.

## Results

### Study characteristics

**Fig. 1:**
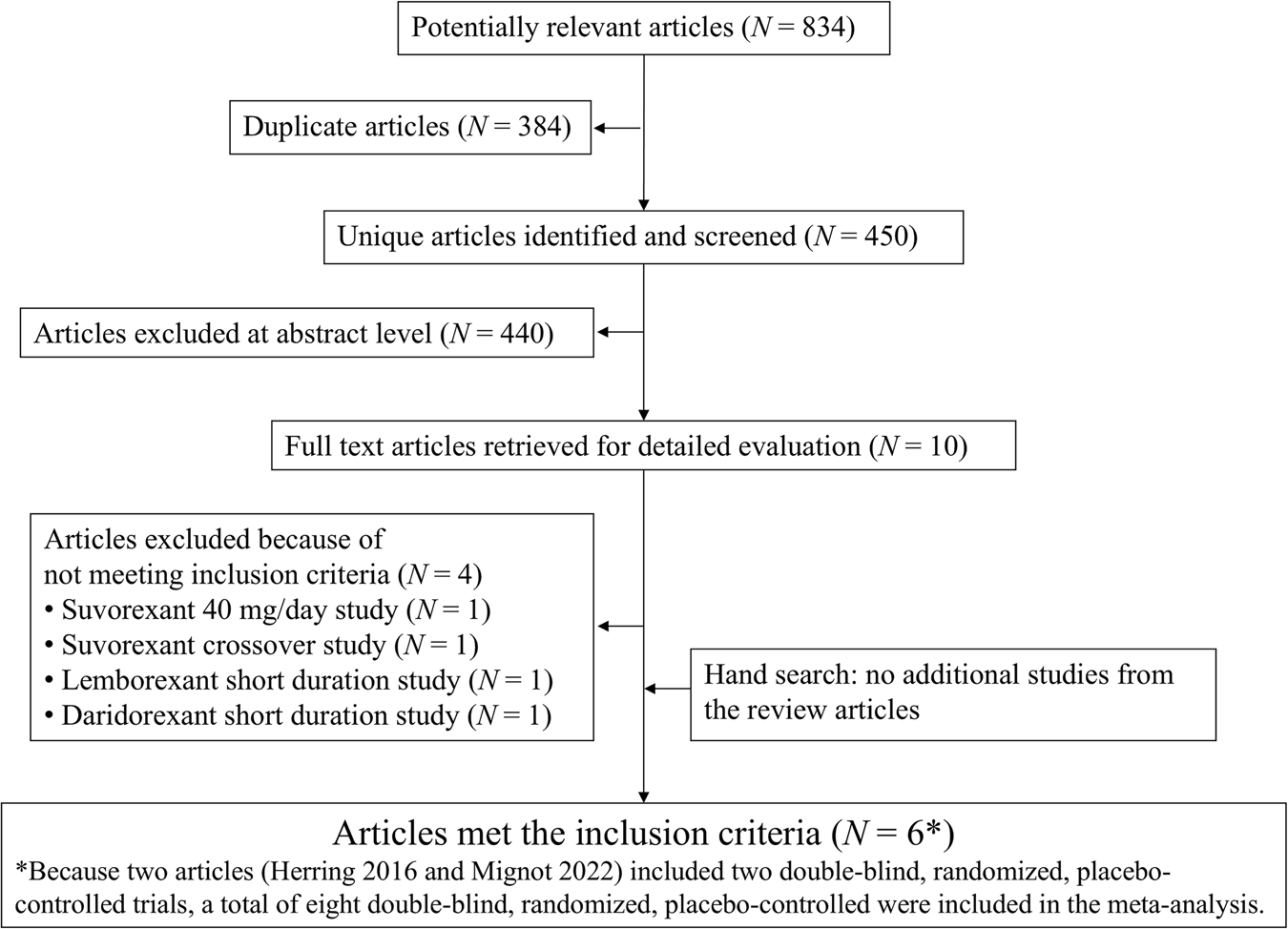
Flow chart of the literature search and study selection.

Figure 1 depicts a flowchart of the literature search, as well as a detailed explanation of the process. Initially, 834 articles were identified, with 384 were duplicates. After title and abstract screening, 440 articles were excluded, with an additional 4 being excluded after full-text review. Previous review articles revealed no additional studies. Finally, six articles were remainded [33, 37–41]. Because two articles included two double-blind, randomized, placebo-controlled trials [38, 41], a total of eight double-blind, randomized, placebo-controlled trials (*n* = 5198, mean age = 56.33 years, with 67.84% females) were included in the meta-analysis. The treatment arms included DAR25, DAR50, LEM5, LEM10, SUV20/15, and placebo. Table 1 summarizes the study characteristics. The diagnostic criteria for all studies included in this systematic review were based on the Diagnostic and Statistical Manual of Mental Disorders [42]. All studies were industry-sponsored. The overall risk of bias in all studies was rated as “Low risk” (Table 2). There was no evidence of violation of the transitivity assumption when comparing study characteristics across different comparisons (Table S2).

**Table 1:** Characteristics of the studies included in the systematic review and network meta-analysis.

Study [1] study name (trial ID), [2] treatment (n), [3] study duration	Inclusion criteria [1] diagnosis, [2] key inclusion criteria, [3] PLA lead-in period	Characteristics of participants [1] mean age (SD), %people≥65 years, [2] %female, [3] race/ethnicity
[1] Dauvilliers [37] (NCT02839200) [2] DAR25 (60), DAR50 (61), PLA (60) [3] 30 days	[1] insomnia disorder (DSM-5) [2] age: 18–64 years, sLSO ≥ 30 min, sWASO ≥ 30 min, and sTST ≤ 6.5 h for ≥ 3 nights/week, and ISI ≥ 15. [3] yes	[1] 45.7 (11.2) years, 0.0% [2] 64.1% [3] Caucasian: 90.6%
[1] Mignot [38] (NCT03545191) [2] DAR25 (310), DAR50 (310), PLA (310) [3] 3 months	[1] insomnia disorder (DSM-5) [2] age ≥ 18 years, sLSO ≥ 30 min, sWASO ≥ 30 min, and sTST ≤ 6.5 h for ≥ 3 nights/week, and ISI ≥ 15. [3] yes	[1] 55.5 (15.3) years, 39.2% [2] 67.1% [3] Caucasian: 90.2%
[1] Mignot [38] (NCT03575104) [2] DAR25 (309), PLA (308) [3] 3 months	[1] insomnia disorder (DSM-5) [2] age ≥ 18 years, sLSO ≥ 30 min, sWASO ≥ 30 min, and sTST ≤ 6.5 h for ≥ 3 nights/week, and ISI ≥ 15. [3] yes	[1] 56.5 (14.2) years, 39.3% [2] 68.6% [3] Caucasian: 87.2%
[1] Uchimura [39] (jRCT2031200452) [2] DAR25 (163), DAR50 (163), PLA (164) [3] 4 weeks	[1] insomnia disorder (DSM-5) [2] age ≥ 18 years, sLSO ≥ 30 min, sWASO ≥ 30 min, and sTST ≤ 6.5 h for ≥ 3 nights/week, and ISI ≥ 15. [3] yes	[1] 54.5 (13.9) years, 30.1% [2] 49.6% [3] Japanese: 100.0%
[1] Rosenberg [40] (NCT02783729) [2] LEM5 (266), LEM10 (269), PLA (208) [3] 1 month	[1] insomnia disorder (DSM-5) [2] male ≥ 65 years and female ≥ 55 years, sWASO ≥ 60 min for ≥ 3 nights/week, ISI ≥ 13, and WASO mean ≥ 60 min on 2 consecutive PSGs with neither night < 45 min. [3] yes	[1] 63.8 (6.7) years, 44.8% [2] 86.5% [3] Caucasian: 74.6%
[1] Kärppä [33] (NCT02952820) [2] LEM 5 (323), LEM 10 (323), PLA (325) [3] 6 months^a^	[1] insomnia disorder (DSM-5) [2] male and female ≥ 18 years, sSOL ≥ 30 min and/or sWASO ≥ 60 min for ≥ 3 nights/week, and ISI ≥ 15. [3] yes	[1] 54.5 (13.8) years, 27.6% [2] 68.2% [3] Caucasian: 71.5%
[1] Herring [41] (NCT01097616) [2] SUV 20/15 (254), PLA (385) [3] 3 months	[1] primary insomnia (DSM-IV-TR) [2] nonelderly (18–65 years) and elderly (≥65 years) patients, sTSO ≥30 min and sTST <6.5 h on ≥ 4 nights/week, and LPS mean >20 min and WASO mean ≥ 60 min on screening and baseline PSG nights with neither night ≤ 45 min. [3] yes	[1] 55.6 (15.4) years, 42.0% [2] 63.8% [3] Caucasian: 64.6%
[1] Herring [41] (NCT01097629) [2] SUV 20/15 (240)^b^, PLA (387) [3] 3 months	[1] primary insomnia (DSM-IV-TR) [2] nonelderly (18–65 years) and elderly ( ≥ 65 years) patients, sTSO ≥ 30 min and sTST < 6.5 h on ≥ 4 nights/week, and LPS mean >20 min and WASO mean ≥ 60 min on screening and baseline PSG nights with neither night ≤ 45 min. [3] yes	[1] 56.6 (15.4) years, 40.5% [2] 65.0% [3] Caucasian: 80.2%

*DAR* daridorexant, *DSM (-TR)* diagnostic and statistical manual of mental disorders (-Text Revision), *ISI* insomnia severity index, *LEM* lemborexant, *LPS* latency to onset of persistent sleep, *n* number of participants, *PLA* placebo, *PSG* polysomnography, *SD* standard deviation, *sSOL* subjective sleep onset latency, *sTSO* subjective time to sleep onset, *sTST* subjective total sleep time, *SUV* suvorexant, (*s)WASO* (subjective) wake-after-sleep onset.

^a^Following the 6-month double-blind randomized placebo-controlled trial (period 1), patients in the placebo group were re-randomized to LEM 5 mg or LEM 10 mg until month 12 (period 2). Therefore, we used data from period 1 in our study.

^b^SUV doses of 20 and 15 mg were administered to nonelderly and elderly patients, respectively.

**Table 2:** Risk of bias summary.

	Randomization process	Deviation from intended intervention	Missing outcome data	Measurement of the outcome	Selection of the reported result	Overall risk of bias
Dauvilliers [37] (NCT02839200)	Low risk	Low risk	Low risk	Low risk	Low risk	Low risk
Mignot [38] (NCT03545191)	Low risk	Low risk	Low risk	Low risk	Low risk	Low risk
Mignot [38] (NCT03575104)	Low risk	Low risk	Low risk	Low risk	Low risk	Low risk
Uchimura [39] (jRCT2031200452)	Low risk	Low risk	Low risk	Low risk	Low risk	Low risk
Rosenberg [40] (NCT02783729)	Low risk	Low risk	Low risk	Low risk	Low risk	Low risk
Kärppä [33] (NCT02952820)	Low risk	Low risk	Low risk	Low risk	Low risk	Low risk
Herring [41] (NCT01097616)	Low risk	Low risk	Low risk	Low risk	Low risk	Low risk
Herring [41] (NCT01097629)	Low risk	Low risk	Low risk	Low risk	Low risk	Low risk

### Network meta-analysis results

#### Efficacy outcomes

**Fig. 2:**
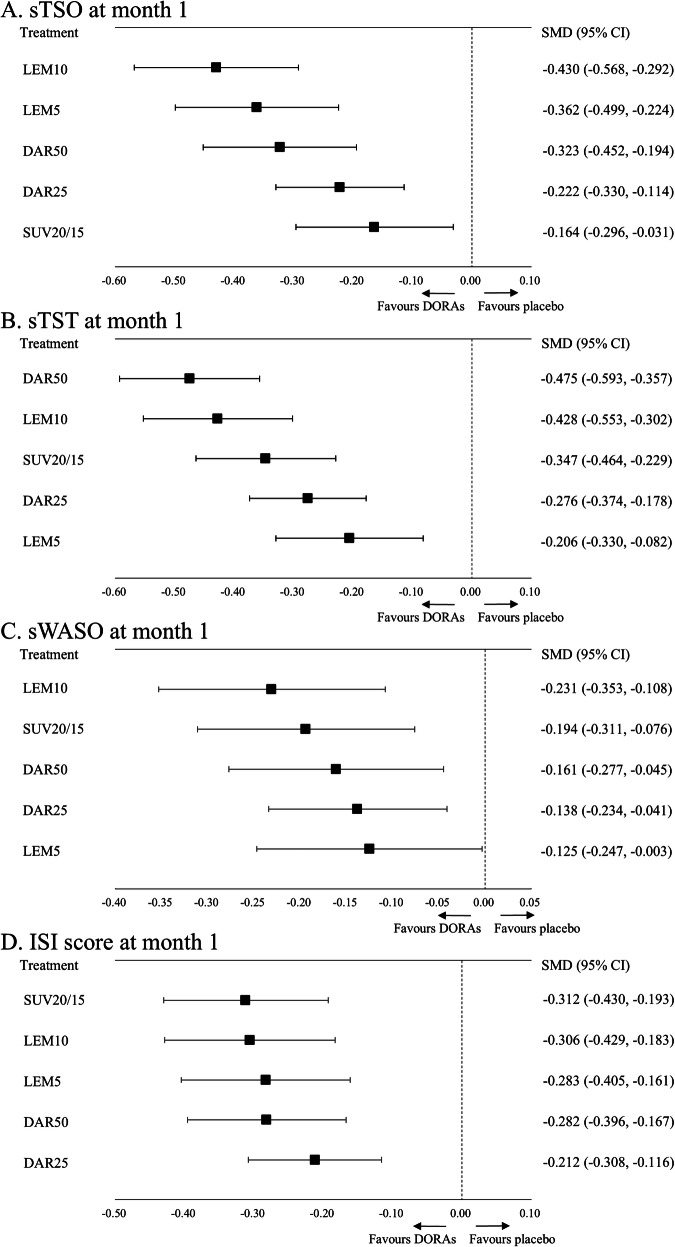
Forest plot. **A** sTSO at month 1. **B** sTST at month 1. **C** sWASO at month 1. **D** ISI score at month 1. 95% CI: 95% confidence interval, DAR25 daridorexant 25 mg/day, DAR50 daridorexant 50 mg/day, ISI insomnia severity index, LEM5 lemborexant 5 mg/day, LEM10 lemborexant 10 mg/day, SMD standardized mean difference, sTSO subjective time to sleep onset, sTST subjective total sleep time, SUV20/15 suvorexant 20 mg/day (15 mg/day for people >65years), sWASO subjective wake after sleep onset.

All active treatments were associated with improvement of sTSO at month 1, sTST at month 1, sWASO at month 1, and ISI scores at month 1 compared to a placebo (Fig. 2, Table 3 and Supplementary Appendix 1–4). The range of standardized mean difference (95% CI) in efficacy outcomes included; sTSO at month 1: −0.430 (−0.568, −0.292) for LEM10 to −0.164 (−0.296, −0.031) for SUV20/15, sTST at month 1: −0.475 (−0.593, −0.357) for DRA50 to −0.206 (−0.330, −0.082) for LEM5, sWASO at month 1: −0.231 (−0.353, −0.108) for LEM10 to −0.125 (−0.247, −0.003) for LEM5, and ISI scores at month 1: −0.312 (−0.430, −0.193) for SUV20/15 to −0.212 (−0.308, −0.116) for DRA25.

**Table 3:** League tables for key efficacy outcomes.

A. sTSO at month 1					
DAR25	0.101 (−0.028, 0.229)	0.139 (−0.036, 0.314)	**0.208 (0.032, 0.383)**	−0.058 (−0.229, 0.113)	−**0.222 (**−**0.330**, −**0.114)**
	DAR50	0.039 (−0.150, 0.227)	0.107 (−0.082, 0.296)	−0.159 (−0.344, 0.026)	−**0.323 (**−**0.452**, −**0.194)**
		LEM5	0.068 (−0.065, 0.202)	−**0.198 (**−**0.389**, −**0.006)**	−**0.362 (**−**0.499**, −**0.224)**
			LEM10	−**0.266 (**−**0.457**, −**0.075)**	−**0.430 (**−**0.568**, −**0.292)**
				SUV20/15	−**0.164 (**−**0.296**, −**0.031)**
					Placebo

B. sTST at month 1					
DAR25	**0.199** (**0.082, 0.316)**	−0.070 (−0.228, 0.088)	0.152 (−0.008, 0.310)	0.071 (−0.082, 0.224)	−**0.276** (−**0.374**, −**0.178)**
	DAR50	−**0.269 (**−**0.440**, −**0.098)**	−0.047 (−0.219, 0.125)	−0.128 (−0.295, 0.038)	−**0.475 (**−**0.593**, −**0.357)**
		LEM5	**0.221 (0.100, 0.342)**	0.141 (−0.030, 0.312)	−**0.206 (**−**0.330**, −**0.082)**
			LEM10	−0.081 (−0.253, 0.091)	−**0.428 (**−**0.553**, −**0.302)**
				SUV20/15	−**0.347 (**−**0.464**, −**0.229)**
					Placebo

C. sWASO at month 1					
DAR25	0.023 (−0.093, 0.140)	−0.013 (−0.168, 0.143)	0.093 (−0.063, 0.249)	0.056 (−0.096, 0.208)	−**0.138 (**−**0.234**, −**0.041)**
	DAR50	−0.036 (−0.205, 0.132)	0.070 (−0.099, 0.239)	0.033 (−0.133, 0.198)	−**0.161 (**−**0.277**, −**0.045)**
		LEM5	0.106 (−0.013, 0.224)	0.069 (−0.100, 0.238)	−**0.125 (**−**0.247**, −**0.003)**
			LEM10	−0.037 (−0.207, 0.132)	−**0.231 (**−**0.353**, −**0.108)**
				SUV20/15	−**0.194 (**−**0.311**, −**0.076)**
					Placebo

D. ISI score at month 1					
DAR25	0.070 (−0.045, 0.184)	0.071 (−0.084, 0.226)	0.094 (−0.062, 0.250)	0.100 (−0.053, 0.252)	−**0.212 (**−**0.308**, −**0.116)**
	DAR50	0.002 (−0.166, 0.169)	0.024 (−0.144, 0.192)	0.030 (−0.135, 0.195)	−**0.282 (**−**0.396**, −**0.167)**
		LEM5	0.023 (−0.096, 0.141)	0.029 (−0.141, 0.199)	−**0.283 (**−**0.405**, −**0.161)**
			LEM10	0.006 (−0.165, 0.177)	−**0.306 (**−**0.429**, −**0.183)**
				SUV20/15	−**0.312 (**−**0.430**, −**0.193)**
					Placebo

Each cell provides the standardized mean difference (SMD) with 95% confidence interval of a comparison. Cells in bold print indicate significant results. Comparisons between treatments should be read from left to right, with the estimate in the cell shared by the column-defining treatment and row-defining treatment. SMDs < 0 prefer the row-defining treatment.

*DAR25* daridorexant 25 mg/day, *DAR50* daridorexant 50 mg/day, *ISI* insomnia severity index, *LEM5* lemborexant 5 mg/day, *LEM10* lemborexant 10 mg/day, *sTSO* subjective time to sleep onset, *sTST* subjective total sleep time, *SUV20/15* suvorexant 20 mg/day (15 mg/day for people ≥65years), *sWASO* subjective wake after sleep onset.

LEM5 was associated with improved sTSO at month 1 compared to SUV20/15 (Table 3 and Supplementary Appendix 1). LEM10 was associated with improved sTSO at month 1 compared to DAR25 and SUV20/15 (Table 3 and Supplementary Appendix 1). DAR50 was associated with improved sTST at month 1 when compared to DAR25 and LEM5 (Table 3 and Supplementary Appendix 2). LEM10 improved sTST at month 1 when compared to LEM5 (Table 3 and Supplementary Appendix 2). The other two efficacy outcomes showed no significant difference between the active treatments (Table 3 and Supplementary Appendix 3–4).

The network meta-regression analyses revealed no potential confounding factors for the effect size of sTSO at month 1 and sTST at month 1 (Supplementary Appendix 1–2).

#### Acceptability, tolerance, and safety outcomes

DRA25, LEM5, LEM10, and SUV20/15 were associated with a higher incidence of somnolence than a placebo (Supplementary Appendix 8), but there were no significant differences in acceptability, tolerability, or other safety outcomes between any active treatments and the placebo (Table 4 and Supplementary Appendix 5–7, 9–13).

**Table 4:** League tables for tolerability and key safety outcomes.

A. Discontinuation due to adverse events^a^					
DAR25	0.969 (0.352, 2.665)	0.815 (0.100, 6.629)	0.548 (0.079, 3.799)	0.958 (0.375, 2.448)	0.639 (0.312, 1.312)
	DAR50	0.841 (0.094, 7.516)	0.565 (0.074, 4.339)	0.988 (0.318, 3.069)	0.660 (0.253, 1.722)
		LEM5	0.672 (0.111, 4.053)	1.175 (0.150, 9.204)	0.784 (0.110, 5.614)
			LEM10	1.749 (0.262, 11.655)	1.167 (0.193, 7.051)
				SUV20/15	0.668 (0.365, 1.221)
					Placebo

B. Somnolence^a^					
DAR25	1.040 (0.542, 1.994)	0.879 (0.230, 3.359)	0.497 (0.137, 1.796)	0.915 (0.393, 2.128)	**1.943 (1.037, 3.640)**
	DAR50	0.845 (0.211, 3.389)	0.478 (0.126, 1.816)	0.880 (0.351, 2.205)	1.868 (0.905, 3.857)
		LEM5	0.565 (0.254, 1.260)	1.041 (0.280, 3.868)	2.211 (0.676, 7.230)
			LEM10	1.842 (0.525, 6.462)	**3.911 (1.274, 12.001)**
				SUV20/15	**2.123 (1.208, 3.734)**
					Placebo

C. Dizziness					
DAR25	0.658 (0.263, 1.649)	2.603 (0.453, 14.972)	3.950 (0.577, 27.040)	1.235 (0.402, 3.792)	1.522 (0.627, 3.694)
	DAR50	3.954 (0.651, 24.011)	5.999 (0.834, 43.155)	1.875 (0.562, 6.256)	2.312 (0.859, 6.218)
		LEM5	1.517 (0.251, 9.153)	0.474 (0.090, 2.488)	0.585 (0.129, 2.641)
			LEM10	0.313 (0.050, 1.969)	0.385 (0.070, 2.124)
				SUV20/15	1.233 (0.620, 2.451)
					Placebo

D. Falls					
DAR25	0.901 (0.148, 5.495)	0.472 (0.067, 3.305)	0.903 (0.121, 6.725)	0.491 (0.065, 3.733)	0.455 (0.118, 1.745)
	DAR50	0.523 (0.059, 4.637)	1.002 (0.107, 9.376)	0.545 (0.057, 5.194)	0.504 (0.095, 2.671)
		LEM5	1.914 (0.431, 8.509)	1.041 (0.131, 8.258)	0.964 (0.236, 3.940)
			LEM10	0.544 (0.065, 4.570)	0.504 (0.113, 2.237)
				SUV20/15	0.926 (0.203, 4.225)
					Placebo

Each cell provides the odd ratio (OR) with 95% confidence interval of a comparison. Cells in bold print indicate significant results. Comparisons between treatments should be read from left to right, with the estimate in the cell shared by the column-defining treatment and row-defining treatment. ORs < 1 support the row-defining treatment.

*DAR25* daridorexant 25 mg/day, *DAR50* daridorexant 50 mg/day, *LEM5* lemborexant 5 mg/day, *LEM10* lemborexant 10 mg/day, *SUV20/15* suvorexant 20 mg/day (15 mg/day for people ≥65years).

^a^The results came from the sensitivity analysis because the primary analysis had considerable heterogeneity.

LEM10 was associated with a higher discontinuation rate due to adverse events than SUV20/15 (Supplementary Appendix 6). LEM10 was linked to a higher incidence of somnolence than DAR25, DAR50, LEM5, and SUV20/15 (Supplementary Appendix 8). Acceptability and other safety outcomes showed no significant differences between active treatments (Supplementary Appendix 5, 7, 9–13).

The sensitivity analysis produced similar results for acceptability, tolerability, and safety outcomes other than discontinuation due to adverse events and somnolence to the primary analysis (Supplementary Appendix 5–13). The sensitivity analysis revealed no significant difference in discontinuation due to adverse events between LEM10 and SUV20/15 (Table 4 and Supplementary Appendix 6). The global heterogeneity of the outcome decreased in the sensitivity analysis compared to the primary analysis (Supplementary Appendix 6). In the sensitivity analysis for the incidence of somnolence, DAR50 and LEM5 did not differ from the placebo, but DAR25, LEM10, and SUV20/15 were again confirmed to be more frequent than the placebo (Table 4 and Supplementary Appendix 8). The sensitivity analysis revealed a substantial reduction in the OR of LEM10 for somnolence compared to the primary analysis (OR [95% CI]: primary analysis = 6.421 [3.145, 13.109], sensitivity analysis 3.911 [1.274, 12.001]). In this sensitivity analysis, the risk of somnolence was not significantly different between LEM10 and any other active treatments (Table 4 and Supplementary Appendix 8). The global heterogeneity of the outcome decreased in the sensitivity analysis compared to the primary analysis (Supplementary Appendix 8).

We did not conduct a network meta-analysis for death, suicidal ideation and/or behavior, sleep paralysis, abnormal dreams, nightmares, or cataplexy because these outcomes are rare (Supplementary Appendix 14).

### Heterogeneity, inconsistency, and network meta-analysis results graded using the CINeMA application

The results for heterogeneity and consistency in all outcomes are depicted in Appendices S1–S13. Global heterogeneity was rated as moderate to high for discontinuation due to adverse events and somnolence. However, the global heterogeneity for these outcomes in the sensitivity analysis, which excluded the long-term study of LEM [33], was substantially reduced, and thus graded low. Global heterogeneity was rated as low to moderate or low for all other outcomes. Although we found considerable local heterogeneity for sTSO at month 1, sTST at month 1, sWASO at month 1, all-cause discontinuation, at least one adverse event, falls, nasopharyngitis, and upper respiratory tract infection in a few specific comparisons, we did not find considerable local heterogeneity for other outcomes. There was no significant global inconsistency across all outcomes. However, we discovered significant local inconsistency when comparing LEM10 vs. placebo for sTSO at month 1, falls, and nasopharyngitis, and LEM5 vs. placebo for sWASO at month 1. The majority of the comparisons’ within-study bias was assessed as having “some concerns.” The funnel plot was symmetrical (Supplementary Appendix 1–2). However, there are only eight available double-blind, randomized, placebo-controlled trials of DORAs in adults with insomnia. Because funnel plots with fewer than 10 studies were unreliable [21], all comparisons for publication bias were rated as “some concerns.” If the comparison only included indirect evidence, it was downgraded one level. Consequently, overall confidence in the evidence was rated generally as low or very low.

## Discussion

DORAs represent a new class of hypnotic agents and are not associated with physiological tolerance, withdrawal, or rebound insomnia when abruptly discontinued, nor are they associated with deleterious effects on sleep architecture [8]. This is the first systematic review and network meta-analysis to compare the efficacy, acceptability, tolerability, and safety of DAR25, DAR50, LEM5, LEM10, SUV20/15, and placebo for adults with insomnia. Our findings showed that all active treatments were expected to improve insomnia symptoms, such as difficulty initiating and maintaining sleep, as well as early morning awakening with the inability to fall back asleep. Furthermore, because there were no significant differences in all-cause discontinuation or discontinuation due to adverse events between any active treatments and placebo, it was concluded that these active treatments were well-accepted and well-tolerated by the people from scientific vantage point. However, clinicians should be aware that DORAs pose a risk of somnolence.

Although all DORAs improved their ISI scores in the first month, there were no significant differences between them. The ISI is a brief instrument designed to assess the severity of both nighttime and daytime components of insomnia, resulting in a total score that can be used to assess the overall sleep quality of people suffering from insomnia [25]. Therefore, any DORAs may be expected to improve overall sleep quality when administered in doses appropriate for each individual with insomnia. Furthermore, we examined the utility of these DORAs in detail by reviewing the results of other efficacy and safety outcomes in which the DORAs differed significantly.

In the first month of sTSO, LEM5 outperformed SUV20/15, while LEM10 outperformed DAR25 and SUV20/15. LEM10 and LEM5 had the first and second largest effect sizes on sTSO at month 1. Thus, LEM is expected to improve the difficulty of initiating sleep, particularly. However, our network meta-analysis revealed that, because DAR50 did not differ from LEM5 and LEM10 in terms of sTSO improvement at month 1, DAR50 may also be a suitable option for people who have difficulty initiating sleep when patients discontinue receiving LEM for various reasons.

For sTST at month 1, DAR50 outperformed DAR25, and LEM10 outperformed LEM5. Thus, DAR and LEM appear to have a stronger effect on sTST in a dose-dependent manner. Furthermore, DAR50 outperformed LEM5 regarding sTST at month 1. DAR50 had the first largest effect size on sTST at month 1. Thus, DAR50 is expected to improve difficulty maintaining sleep, in particular. However, LEM10 had the second largest effect size on sTST at month 1. Therefore, LEM may be a good option for adults who struggle to fall and stay asleep. Although all DORAs improved WASO at month 1, which is considered another outcome for maintaining sleep, there were no significant differences in the outcome between these DORAs.

In our network meta-analysis, while all DORAs did not differ from placebo in terms of all-cause discontinuation rate, discontinuation rate due to adverse events, or incidence of at least one adverse event, all DORAs except DAR50 were associated with somnolence. For global heterogeneity in somnolence, because “moderate to high” was evaluated in the primary analysis and “low” was evaluated in the sensitivity analysis, the sensitivity analysis result was deemed more confident than the primary analysis. Therefore, we discuss the risk of somnolence based on the results of the sensitivity analysis. In the sensitivity analysis, while DAR25, LEM10, and SUV20/15 were associated with a higher incidence of somnolence than placebo, there were no significant differences in the incidence of somnolence between any DORAs. LEM5 did not differ from placebo in terms of somnolence incidence, and LEM appeared to have a higher risk in a dose-dependent manner. However, our network meta-analysis revealed that DAR25, but not DAR50, increased the risk of somnolence. Because DAR50 has greater efficacy regarding insomnia than DAR25, DAR50 may have reduced the risk of daytime sleepiness associated with insomnia more than that observed with DAR25. In our network meta-analysis, the first and second lowest ORs for somnolence were DAR50 and DAR25. DAR appears to have the lowest risk of somnolence among DORAs. DAR has a shorter half-life after repeated doses than LEM and SUV [6, 43–45], which could explain why DAR had a lower incidence of somnolence. According to individual DAR studies [37–39], the incidences of somnolence in the DAR50, DAR25, and placebo arms were 1.62–6.79%, 3.25–6.67%, and 1.31–5.00%, respectively. Thus, while our network meta-analysis did not identify a risk of somnolence for DAR50, clinicians and insomnia patients should exercise caution even when using DAR50.

Nonetheless, the Japan study found that DAR50 (6.79%) may be associated with a higher incidence of somnolence than DAR25 (3.68%) [39]. Other studies with different populations did not find this difference [37, 38]. The primary P450 enzyme involved in the metabolism of DAR was CYP3A4, which accounted for 89% of metabolic turnover [46]. According to the review [47], CYP3A4 activity varies by 10–100 fold between individuals. However, the cause of this has not been fully explained in terms of genetic variation [48], and there were no common polymorphisms in the CYP3A4 gene that are not only involved in the metabolism of CYP3A4 activity but also specific to the Japanese population [49]. Nonetheless, a recent study using individual data from a validated consumer sleep wearable device from over 50 million nights of sleep in over 220,000 people from 35 countries found that nocturnal sleep was shorter and started later in Asia, including Japan, than in other regions [50]. DAR’s pharmacokinetic study found that the area under the plasma concentration–time curve increased dose-dependently from 0–24 h [51] Therefore, administering DAR50 to Japanese individuals with short sleep duration may have increased the likelihood of a carryover effect the next morning. However, the Japan phase 3 study of DAR found that the effects were achieved without an increase in morning sleepiness and rather, the Visual Analog Scale score for morning sleepiness improved in the DAR groups [39]. Thus, we could not conduct a thorough discussion of the relationship between DAR and the risk of somnolence in Japan.

A recent meta-analysis found that benzodiazepines are consistently associated with an increased risk of falls in elderly people [52]. Furthermore, another meta-analysis found that benzodiazepines are associated with an increased risk of dizziness or light-headedness in the people with insomnia [53]. However, our network meta-analysis revealed that all DORAs were not associated with an increased risk of falls and dizziness in people with insomnia.

Compared with other published network meta-analyses on the topic [22–24], the strengths of our network meta-analysis were as follows; First, our network meta-analysis has added the most recent trial of DAR [39]. Second, our network meta-analysis included the important safety outcomes such as somnolence, dizziness, and falls. Moreover, we performed a sensitivity analysis using data of acceptability, tolerability, and safety outcomes, where observation periods could be matched. Therefore, the results of our network meta-analysis may currently provide the most relevant answer to the clinical question of which DORAs are better in terms of efficacy, acceptability, tolerability, and safety for adult individuals with insomnia disorder.

Our study had several limitations. First, our meta-analysis included a relatively small number of participants and studies. Second, the studies included in our meta-analysis had short durations. Third, we discovered “considerable” global heterogeneity among some safety outcomes; however, the global heterogeneity for the sensitivity analysis in these outcomes was substantially reduced. A possible reason for the considerable heterogeneity in the outcomes of the primary analysis was determined to be related to the inclusion of data from a long-term study for LEM in the network meta-analysis [33]. Fourth, because we did not combine low-dose and high-dose treatment arms for LEM or DAR, we did not account for the unit of analysis error (resulting in overly precise results) in our meta-analysis as described in the Cochrane Handbook [21]. Fifth, our meta-analysis did not include the outcomes related to polysomnography because there was insufficient data to do so. Sixth, our study did not address several aspects of making informed decisions in everyday clinical practice, such as integration with pharmacotherapy, other nonpharmacological interventions, and cost-effectiveness analysis. Ultimately, understanding differences and similarities among the DORAs and other hypnotics would be enhanced by the conduct of adequately powered, randomized, controlled head-to-head clinical trials, so that direct comparisons can be made.

## Supplementary information

Supplementary material

## Data Availability

Data used for the current study were reported in articles as cited in this paper.
