# Supplementary material for: Comparative efficacy and safety of daridorexant, lemborexant, and suvorexant for insomnia: a systematic review and network meta-analysis
Source: Transl Psychiatry. 2025 Jun 24;15:211. doi: 10.1038/s41398-025-03439-8 (PMC12187915; doi:10.1038/s41398-025-03439-8)
Supplement: Supplementary file 1 — Supplementary material [file 41398_2025_3439_MOESM1_ESM.docx]

**Table S1. PRISMA for Network Meta-Analyses Checklist.**

| **Section/Topic** | **Item #** | **Checklist Item** | **Reported on Page #** |
| --- | --- | --- | --- |
| **TITLE** |  |  |  |
| Title | 1 | Identify the report as a systematic review *incorporating a network meta-analysis (or related form of meta-analysis).* | 1 |
| **ABSTRACT** |  |  |  |
| Structured summary | 2 | Provide a structured summary including, as applicable:  **Background:** main objectives  **Methods:** data sources; study eligibility criteria, participants, and interventions; study appraisal; and *synthesis methods, such as network meta-analysis.*  **Results:** number of studies and participants identified; summary estimates with corresponding confidence/credible intervals; *treatment rankings may also be discussed. Authors may choose to summarize pairwise comparisons against a chosen treatment included in their analyses for brevity.*  **Discussion/Conclusions:** limitations; conclusions and implications of findings.  **Other:** primary source of funding; systematic review registration number with registry name. | 3- |
| **INTRODUCTION** |  |  |  |
| Rationale | 3 | Describe the rationale for the review in the context of what is already known*, including mention of why a network meta-analysis has been conducted.* | 5- |
| Objectives | 4 | Provide an explicit statement of questions being addressed, with reference to participants, interventions, comparisons, outcomes, and study design (PICOS). | 5- |
| **METHODS** |  |  |  |
| Protocol and registration | 5 | Indicate whether a review protocol exists and if and where it can be accessed (e.g., Web address); and, if available, provide registration information, including registration number. | 7- |
| Eligibility criteria | 6 | Specify study characteristics (e.g., PICOS, length of follow-up) and report characteristics (e.g., years considered, language, publication status) used as criteria for eligibility, giving rationale. *Clearly describe eligible treatments included in the treatment network, and note whether any have been clustered or merged into the same node (with justification).* | 7- |
| Information sources | 7 | Describe all information sources (e.g., databases with dates of coverage, contact with study authors to identify additional studies) in the search and date last searched. | 7- |
| Search | 8 | Present full electronic search strategy for at least one database, including any limits used, such that it could be repeated. | 7- |
| Study selection | 9 | State the process for selecting studies (i.e., screening, eligibility, included in systematic review, and, if applicable, included in the meta-analysis). | 7- |
| Data collection process | 10 | Describe method of data extraction from reports (e.g., piloted forms, independently, in duplicate) and any processes for obtaining and confirming data from investigators. | 7- |
| Data items | 11 | List and define all variables for which data were sought (e.g., PICOS, funding sources) and any assumptions and simplifications made. | 7- |
| **Geometry of the network** | **S1** | Describe methods used to explore the geometry of the treatment network under study and potential biases related to it. This should include how the evidence base has been graphically summarized for presentation, and what characteristics were compiled and used to describe the evidence base to readers. | 7- |
| Risk of bias within individual studies | 12 | Describe methods used for assessing risk of bias of individual studies (including specification of whether this was done at the study or outcome level), and how this information is to be used in any data synthesis. | 7- |
| Summary measures | 13 | State the principal summary measures (e.g., risk ratio, difference in means). *Also describe the use of additional summary measures assessed, such as treatment rankings and surface under the cumulative ranking curve (SUCRA)* values, as well as modified approaches used to present summary findings from meta-analyses.* | 7- |
| Planned methods of analysis | 14 | Describe the methods of handling data and combining results of studies for each network meta-analysis. This should include, but not be limited to:   - *Handling of multi-arm trials;* - *Selection of variance structure;* - *Selection of prior distributions in Bayesian analyses; and* - *Assessment of model fit.* | 7- |
| **Assessment of Inconsistency** | **S2** | Describe the statistical methods used to evaluate the agreement of direct and indirect evidence in the treatment network(s) studied. Describe efforts taken to address its presence when found. | 7- |
| Risk of bias across studies | 15 | Specify any assessment of risk of bias that may affect the cumulative evidence (e.g., publication bias, selective reporting within studies). | 7- |
| Additional analyses | 16 | Describe methods of additional analyses if done, indicating which were pre-specified. This may include, but not be limited to, the following:   - Sensitivity or subgroup analyses; - Meta-regression analyses; - *Alternative formulations of the treatment network; and* - *Use of alternative prior distributions for Bayesian analyses (if applicable).* | 7- |
| **RESULTS†** |  |  |  |
| Study selection | 17 | Give numbers of studies screened, assessed for eligibility, and included in the review, with reasons for exclusions at each stage, ideally with a flow diagram. | 10- |
| **Presentation of network structure** | **S3** | Provide a network graph of the included studies to enable visualization of the geometry of the treatment network. | 10- |
| **Summary of network geometry** | **S4** | Provide a brief overview of characteristics of the treatment network. This may include commentary on the abundance of trials and randomized patients for the different interventions and pairwise comparisons in the network, gaps of evidence in the treatment network, and potential biases reflected by the network structure. | 10- |
| Study characteristics | 18 | For each study, present characteristics for which data were extracted (e.g., study size, PICOS, follow-up period) and provide the citations. | 10- |
| Risk of bias within studies | 19 | Present data on risk of bias of each study and, if available, any outcome level assessment. | 10- |
| Results of individual studies | 20 | For all outcomes considered (benefits or harms), present, for each study: 1) simple summary data for each intervention group, and 2) effect estimates and confidence intervals. *Modified approaches may be needed to deal with information from larger networks.* | 10- |
| Synthesis of results | 21 | Present results of each meta-analysis done, including confidence/credible intervals. *In larger networks, authors may focus on comparisons versus a particular comparator (e.g. placebo or standard care), with full findings presented in an appendix. League tables and forest plots may be considered to summarize pairwise comparisons.* If additional summary measures were explored (such as treatment rankings), these should also be presented. | 10- |
| **Exploration for inconsistency** | **S5** | Describe results from investigations of inconsistency. This may include such information as measures of model fit to compare consistency and inconsistency models, *P* values from statistical tests, or summary of inconsistency estimates from different parts of the treatment network. | 10- |
| Risk of bias across studies | 22 | Present results of any assessment of risk of bias across studies for the evidence base being studied. | 10- |
| Results of additional analyses | 23 | Give results of additional analyses, if done (e.g., sensitivity or subgroup analyses, meta-regression analyses*, alternative network geometries studied, alternative choice of prior distributions for Bayesian analyses,* and so forth). | 10- |
| **DISCUSSION** |  |  |  |
| Summary of evidence | 24 | Summarize the main findings, including the strength of evidence for each main outcome; consider their relevance to key groups (e.g., healthcare providers, users, and policy-makers). | 14- |
| Limitations | 25 | Discuss limitations at study and outcome level (e.g., risk of bias), and at review level (e.g., incomplete retrieval of identified research, reporting bias). *Comment on the validity of the assumptions, such as transitivity and consistency. Comment on any concerns regarding network geometry (e.g., avoidance of certain comparisons).* | 14- |
| Conclusions | 26 | Provide a general interpretation of the results in the context of other evidence, and implications for future research. | 14- |
|  |  |  |  |
| **FUNDING** |  |  | 19 |
| Funding | 27 | Describe sources of funding for the systematic review and other support (e.g., supply of data); role of funders for the systematic review. This should also include information regarding whether funding has been received from manufacturers of treatments in the network and/or whether some of the authors are content experts with professional conflicts of interest that could affect use of treatments in the network. |  |

**Table S2. Transitivity assessment.**

|  | Boxplot | Kruskal–Wallis equality of populations rank test. |
| --- | --- | --- |
| Proportion of females (k = 8) | 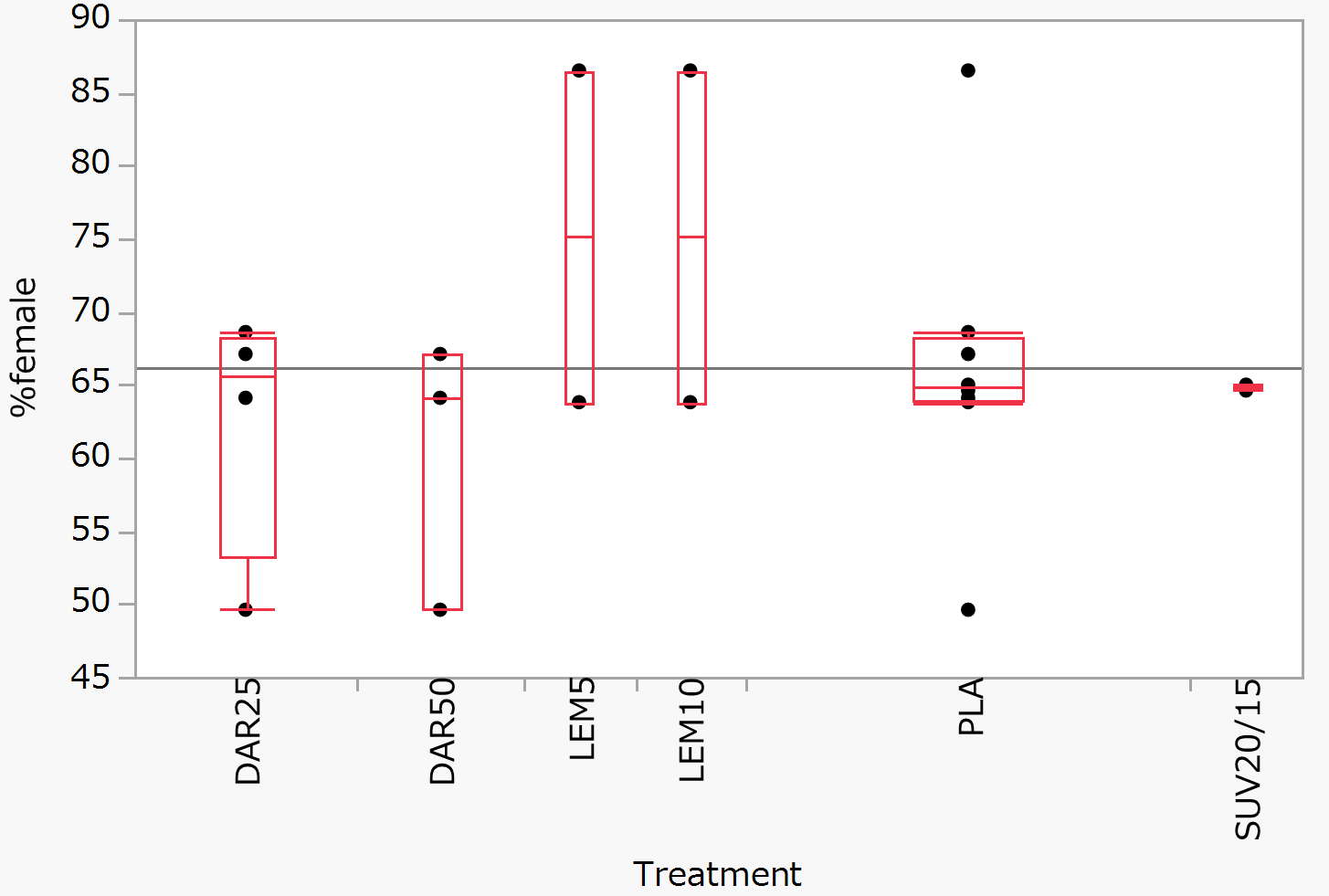 | Chi-squared with ties = 0.85 (df = 5), p = 0.974 |
| Mean age (k = 8) | 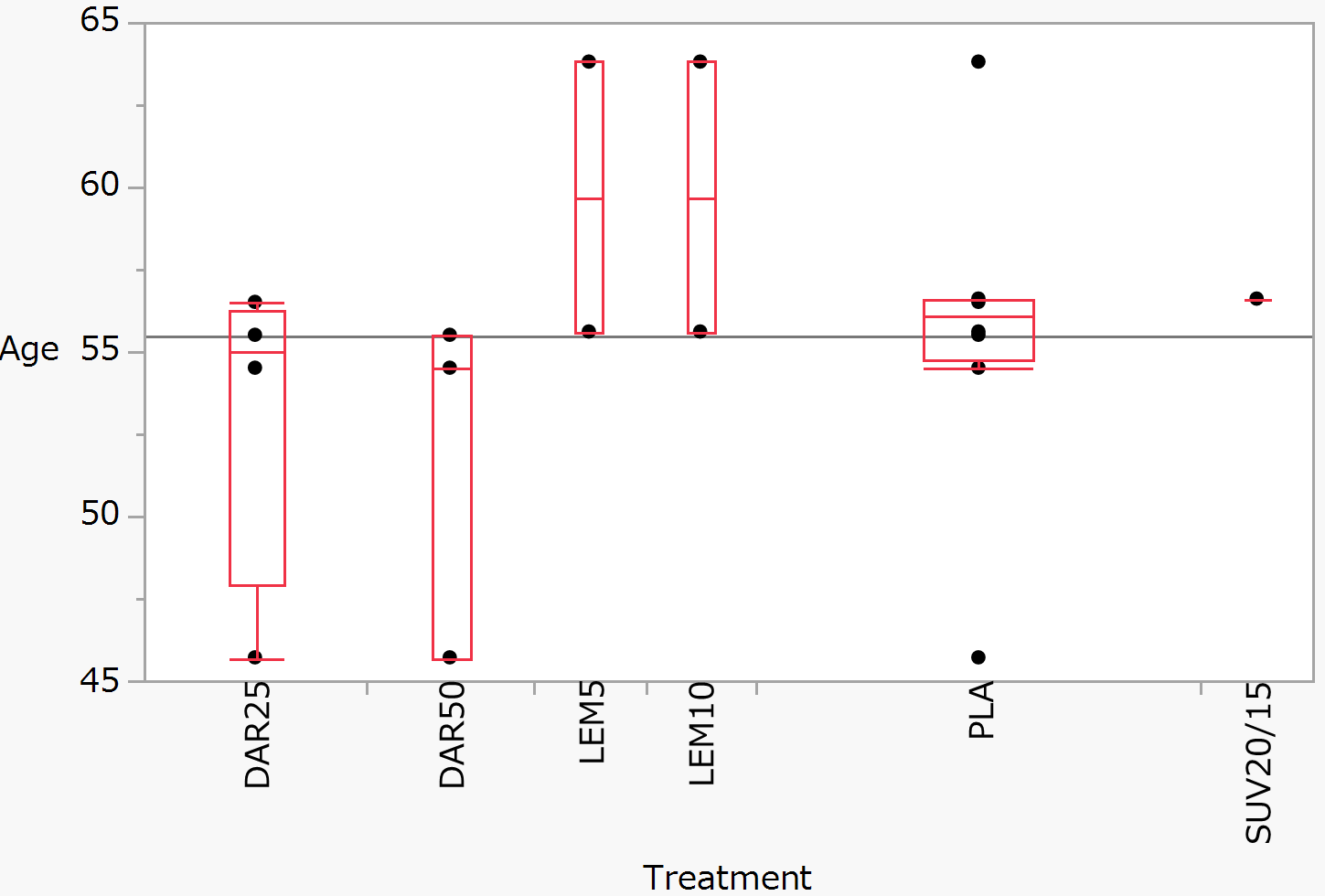 | Chi-squared with ties = 8.27 (df = 5), p = 0.142 |
| Proportion of elderly people (aged >65 years) (k = 8) | 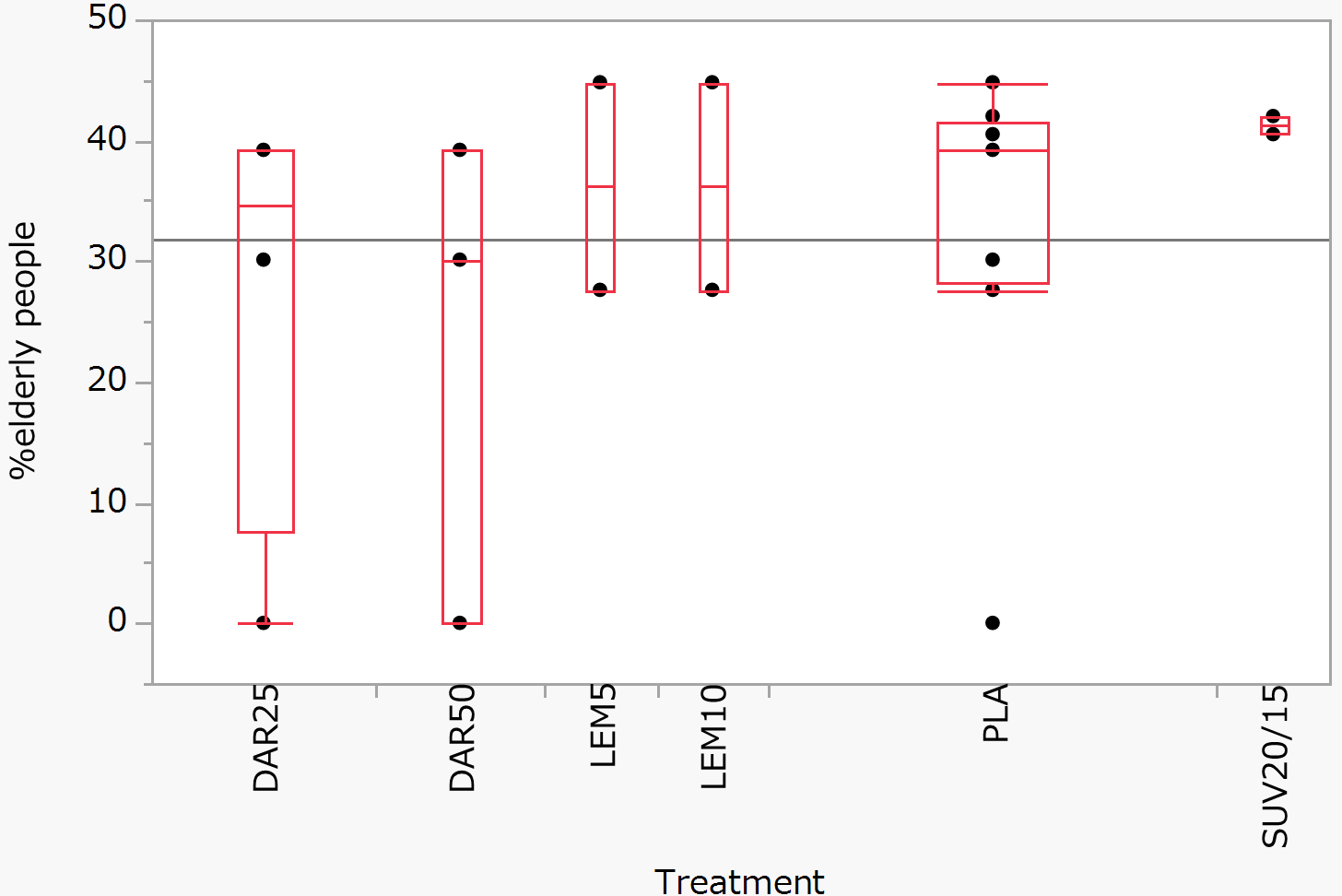 | Chi-squared with ties = 3.76 (df = 5), p = 0.585 |
| Total number of participants (k = 8) | 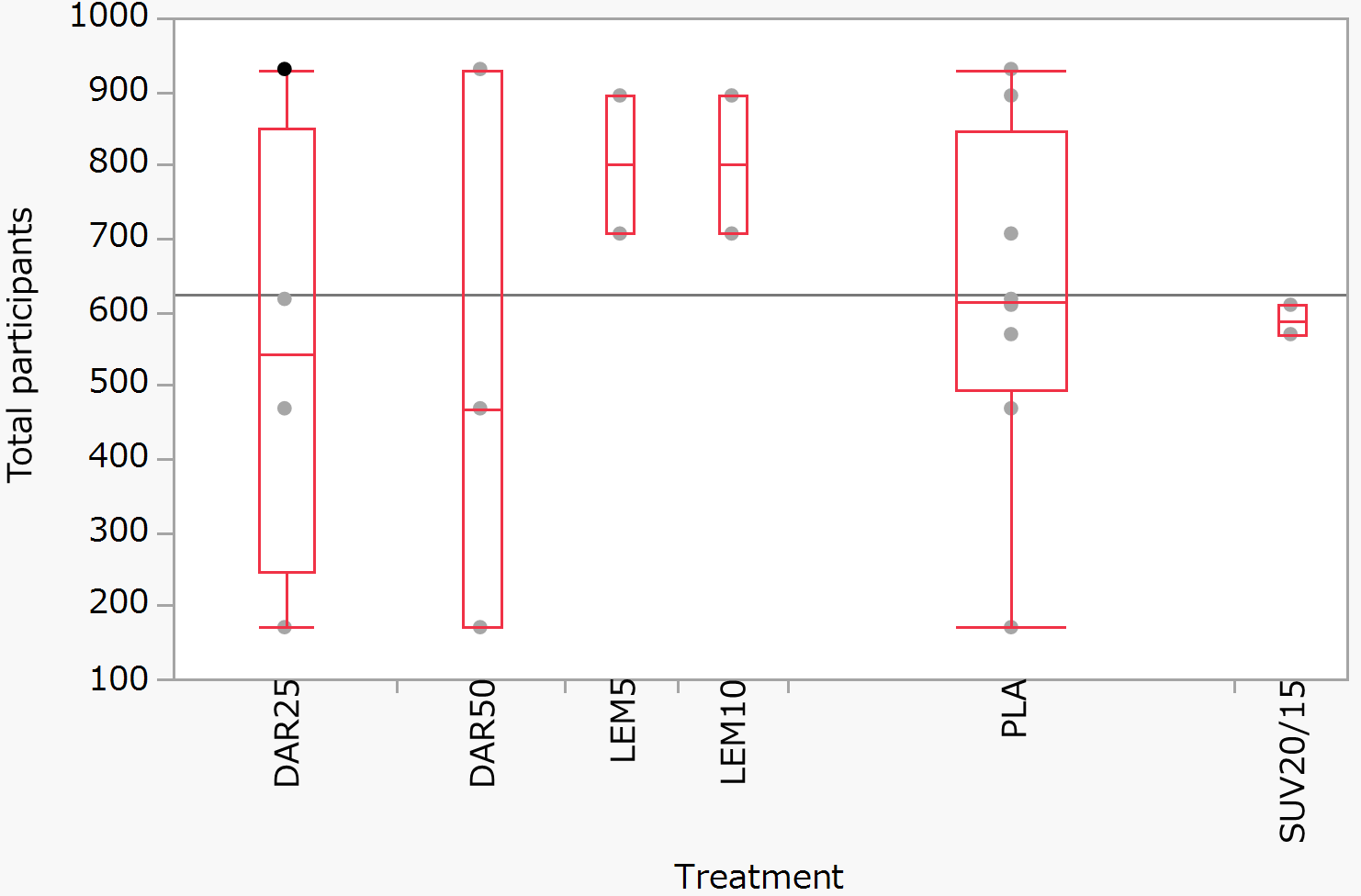 | Chi-squared with ties = 2.99 (df = 5), p = 0.702 |
| Publication year (k = 8) | 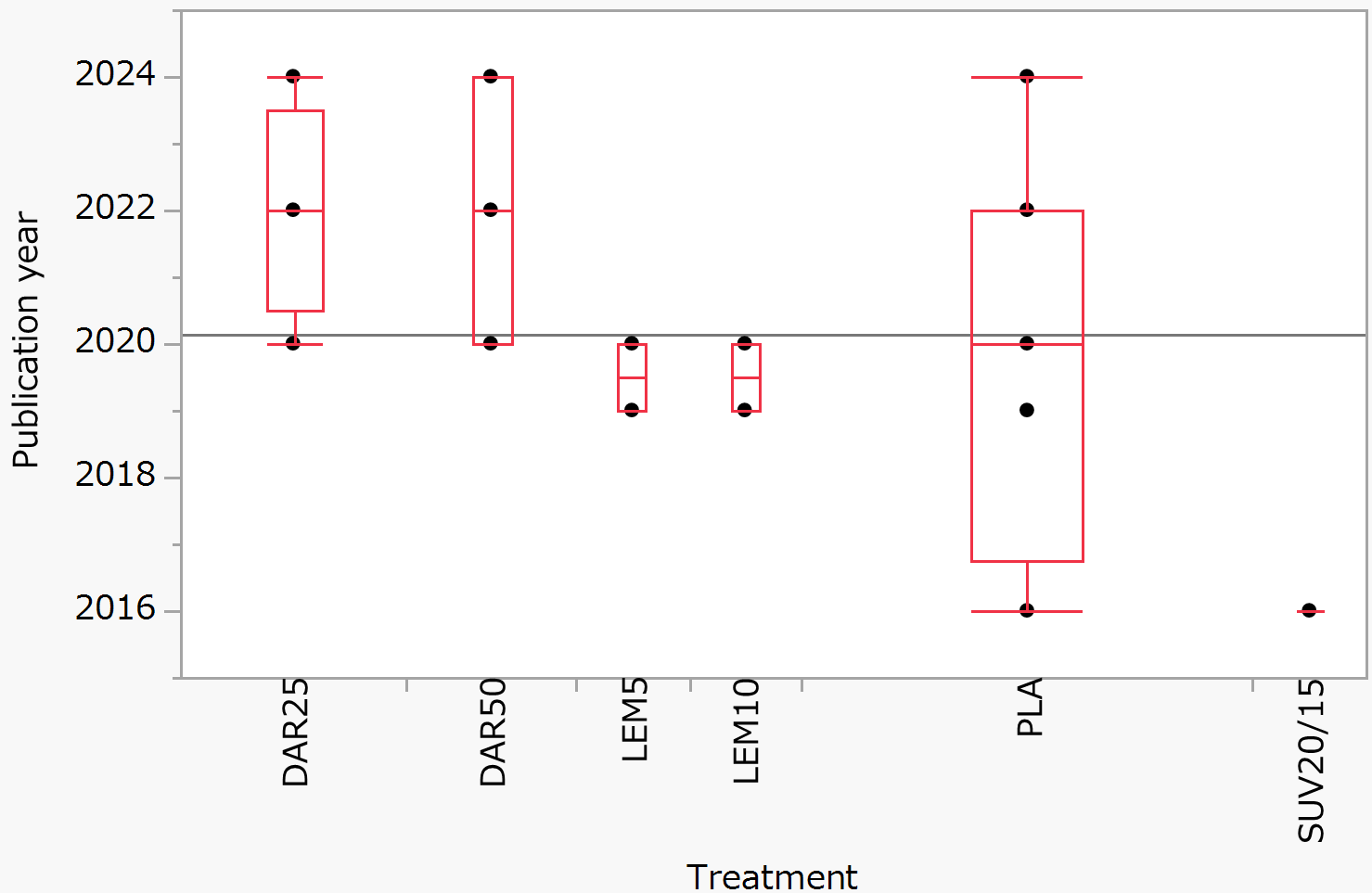 | Chi-squared with ties = 8.81 (df = 5), p = 0.117 |

**Appendix S1. Subjective time to sleep onset at month 1**


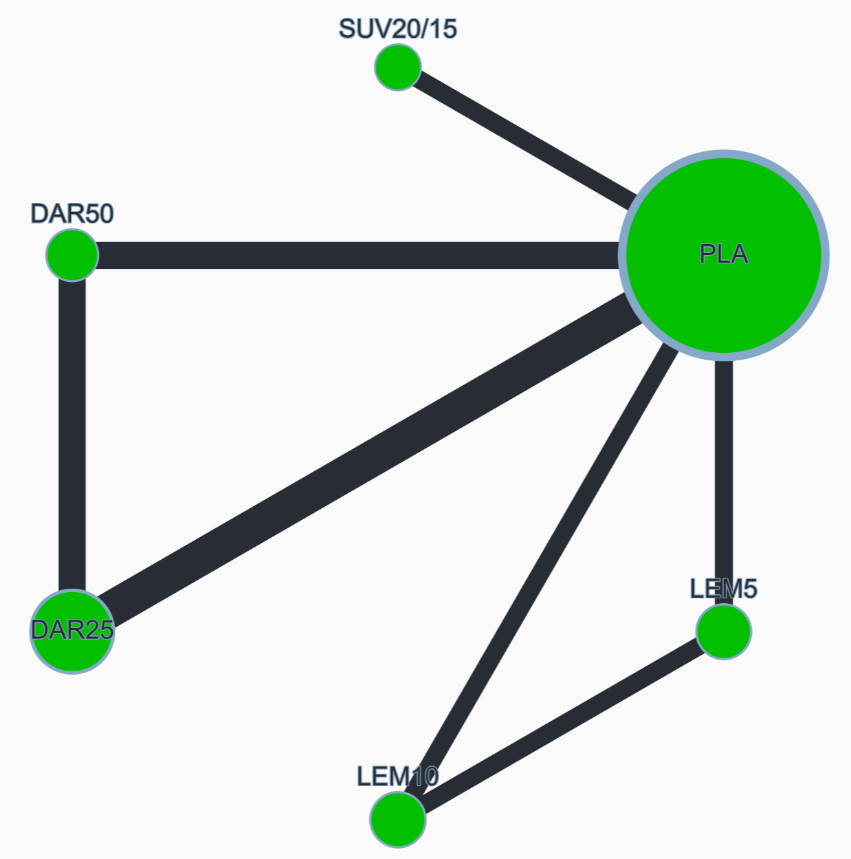
8 studies, 4963 participants

Node size by sample size

Node color by risk of bias

Green: low overall risk of bias

Edge width by number of studies

DAR25: daridorexant 25 mg/day

DAR50: daridorexant 50 mg/day

LEM5: lemborexant 5 mg/day

LEM10: lemborexant 10 mg/day

SUV20/15: suvorexant 20 mg/day (15 mg/day for people >65years)

PLA: placebo

**League table (SMD with 95% CI)**

| DAR25 | 0.101 (-0.028, 0.229) | 0.139 (-0.036, 0.314) | **0.208 (0.032, 0.383)** | -0.058 (-0.229, 0.113) | **-0.222 (-0.330, -0.114)** |
| --- | --- | --- | --- | --- | --- |
|  | DAR50 | 0.039 (-0.150, 0.227) | 0.107 (-0.082, 0.296) | -0.159 (-0.344, 0.026) | **-0.323 (-0.452, -0.194)** |
|  |  | LEM5 | 0.068 (-0.065, 0.202) | **-0.198 (-0.389, -0.006)** | **-0.362 (-0.499, -0.224)** |
|  |  |  | LEM10 | **-0.266 (-0.457, -0.075)** | **-0.430 (-0.568, -0.292)** |
|  |  |  |  | SUV20/15 | **-0.164 (-0.296, -0.031)** |
|  |  |  |  |  | PLA |

**Global heterogeneity**

As previously suggested (Huhn 2019), the common 𝜏^2^ was compared to the empirical distributions of heterogeneity found in the meta‐analyses of pharmacological treatments for mental health outcomes, with a median of the 𝜏^2^ distribution of 0.049 and an inter‐quartile range of 0.010 to 0.242 (Rhodes 2015), and the heterogeneity was considered low when the estimated 𝜏^2^ was below the 25% quartile, moderate when between 25% and 50% of the quartile, and high when above the 50% quartile.

Huhn M, et al. Lancet 2019;394(10202):939-51

Rhodes KM, et al. J Clin Epidemiol 2015;68(1):52-60

Between study variance (𝜏^2^): 0.002 (heterogeneity assessment: low)

**Random-effects design-by-treatment interaction model**

p < 0.1 was considered as considerable global inconsistency.

χ^2^ statistic: 0.012 (1 degrees of freedom), P value: 0.912

**Local heterogeneity (I^2^) and incoherence (SIDE test)**

I^2^ > 50% was considered as considerable heterogeneity.

As a general rule, there are “no concerns” if the p-value is >0.10, independent of the position of the 95% CIs with respect to the range of equivalence, because the evidence for incoherence is weak (p > 0.10).

Nikolakopoulou A, et al., PLOS Medicine 2020 17 1-19, Papakonstantinou T, et al., Campbell Systematic Reviews 2020 16 e1080

|  | NMA SMD | Direct SMD | I^2^ | Indirect SMD | P value (SIDE test) |
| --- | --- | --- | --- | --- | --- |
| DAR25 vs DAR50 | 0.101 (-0.028, 0.229) | 0.098 (-0.037, 0.233) | 32.2% | 0.131 (-0.302, 0.564) | 0.221 |
| DAR25 vs PLA |  | -0.222(-0.330, -0.114) | 0.0% |  |  |
| DAR50 vs PLA | -0.323 (-0.452, -0.194) | -0.327 (-0.462, -0.191) | 0.0% | -0.287 (-0.717, 0.143) | 0.863 |
| LEM5 vs LEM10 |  | 0.068(-0.066, 0.201) | 36.5% |  |  |
| LEM5 vs PLA | -0.361 (-0.499, -0.224) | -0.361 (-0.499, -0.223) | **80.2%** | -0.703 (-4.792, 3.386) | 0.870 |
| LEM10 vs PLA | -0.430 (-0.568, -0.292) | -0.435 (-0.573, -0.297) | 10.5% | 2.726 (-0.742, 6.193) | **0.074** |
| SUV20/15 vs PLA |  | -0.164(-0.296, -0.031) | 0.0% |  |  |

**Funnel plot (all drugs vs. placebo)**


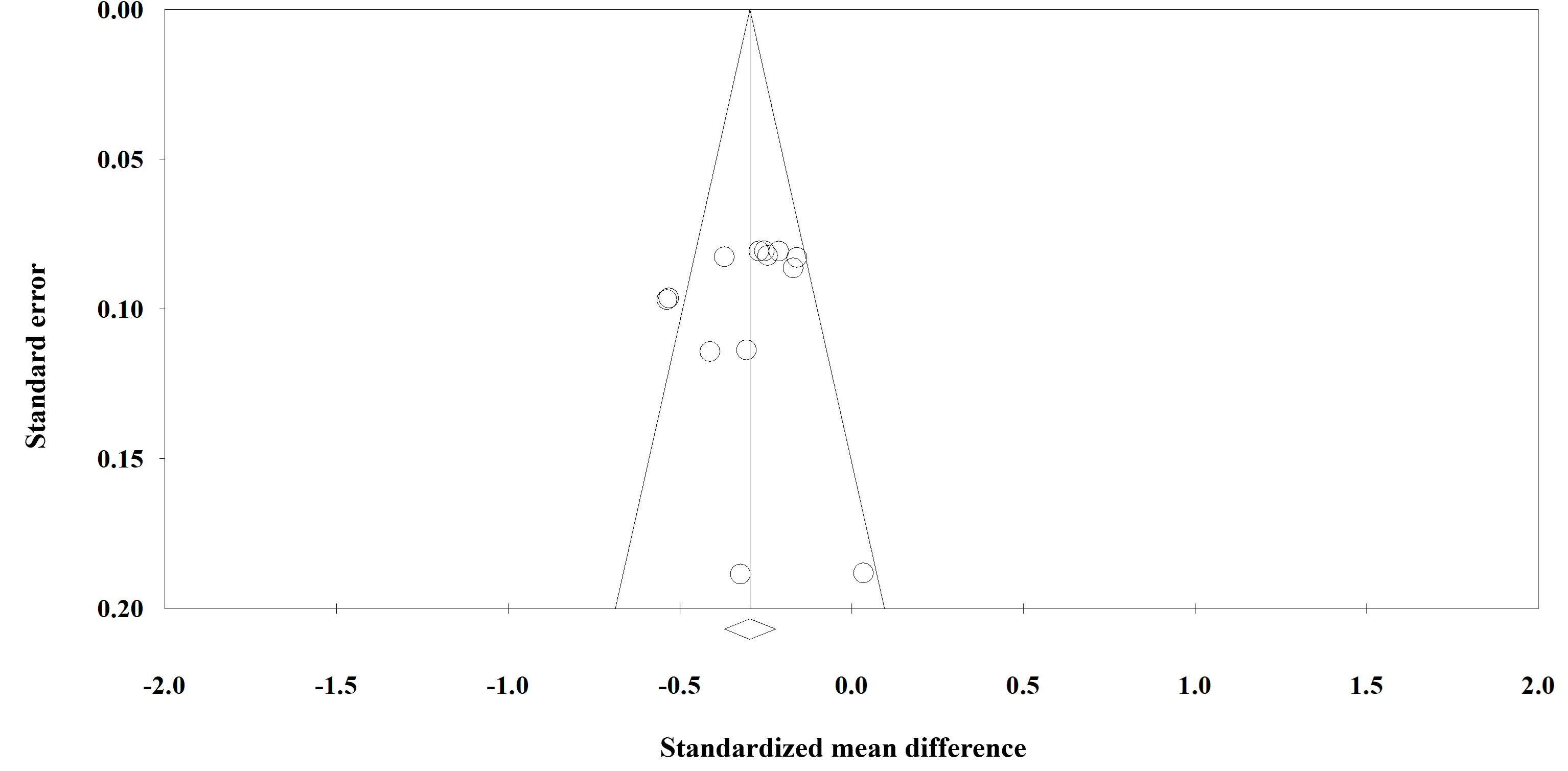


**Meta-regression analysis (the placebo was the control)**

| Potential moderators | β (95% CrI) |
| --- | --- |
| Mean age (k = 8) | 0.274 (-31.169, 36.722) |
| Proportion of females (k = 8) | -0.166 (-25.486, 0.132) |
| Proportion of elderly patients (k = 8) | -0.108 (-21.259, 45.141) |
| Publication year (k = 8) | 0.141 (-67.445, 56.834) |
| Total participants (k = 8) | -0.272 (-32.257, 0.217) |

**CINeMA confidence rating**

CINeMA is a web application that simplifies the evaluation of confidence in the findings from a network meta-analysis. CINeMA is based on a methodological framework described in the following articles, which consider the following six domains: within-study bias, reporting bias, indirectness, imprecision, heterogeneity, and incoherence. CINeMA grades the confidence in the results of each treatment comparison as high, moderate, low, or very low. If the comparison had only indirect evidence, the comparison was downgraded one level.

Nikolakopoulou A, et al., PLOS Medicine 2020 17 1-19, Papakonstantinou T, et al., Campbell Systematic Reviews 2020 16 e1080

(1) Within-study bias: Risk of bias in RCTs for the main outcomes was assessed independently using the Cochrane risk-of-bias tool for randomized trials (RoB 2). In all studies, the overall risk of bias was evaluated as “low risk.” Selected rule: Average

(2) Reporting bias: Comparison-adjusted funnel plots with less than 10 studies are not meaningful. Therefore, all comparisons were “Suspected.”

(3) Indirectness: No indirectness was assumed. Selected rule: Average

(4) Imprecision: For placebo comparisons the clinically meaningful threshold was set at a OR of higher or lower than 1. For placebo comparisons the clinically meaningful threshold was set at a SMD of higher or lower than 0. For comparisons of two active treatments the clinically meaningful threshold was set at SMDs of -0.1 and 0.1, and at ORs of 0.8 and 1.25

for dichotomous outcomes.

(5) Heterogeneity: We used recommendations automatically provided by CINeMA.

(6) Incoherence: We used recommendations automatically provided by CINeMA.

| Comparison | Number of studies | Within-study bias | Reporting bias | Indirectness | Imprecision | Heterogeneity | Incoherence | Confidence rating |
| --- | --- | --- | --- | --- | --- | --- | --- | --- |
| DAR25 vs DAR50 | 3 | No concerns | Some concerns | No concerns | Major concerns | No concerns | No concerns | Low |
| DAR25 vs PLA | 4 | No concerns | Some concerns | No concerns | No concerns | No concerns | No concerns | Moderate |
| DAR50 vs PLA | 3 | No concerns | Some concerns | No concerns | No concerns | No concerns | No concerns | Moderate |
| LEM5 vs LEM10 | 2 | No concerns | Some concerns | No concerns | Major concerns | No concerns | No concerns | Low |
| LEM5 vs PLA | 2 | No concerns | Some concerns | No concerns | No concerns | No concerns | No concerns | Moderate |
| LEM10 vs PLA | 2 | No concerns | Some concerns | No concerns | No concerns | No concerns | Major concerns | Low |
| SUV20/15 vs PLA | 2 | No concerns | Some concerns | No concerns | No concerns | Major concerns | No concerns | Low |
| DAR25 vs LEM5 | 0 | No concerns | Some concerns | No concerns | Major concerns | No concerns | No concerns | High |
| DAR25 vs LEM10 | 0 | No concerns | Some concerns | No concerns | No concerns | Major concerns | No concerns | High |
| DAR25 vs SUV20/15 | 0 | No concerns | Some concerns | No concerns | Major concerns | No concerns | No concerns | High |
| DAR50 vs LEM5 | 0 | No concerns | Some concerns | No concerns | Major concerns | No concerns | No concerns | High |
| DAR50 vs LEM10 | 0 | No concerns | Some concerns | No concerns | Major concerns | No concerns | No concerns | High |
| DAR50 vs SUV20/15 | 0 | No concerns | Some concerns | No concerns | Major concerns | No concerns | No concerns | High |
| LEM5 vs SUV20/15 | 0 | No concerns | Some concerns | No concerns | No concerns | Major concerns | No concerns | High |
| LEM10 vs SUV20/15 | 0 | No concerns | Some concerns | No concerns | No concerns | No concerns | No concerns | Low |

**Appendix S2.** **Subjective total sleep time at month 1**

8 studies, 4855 participants

**
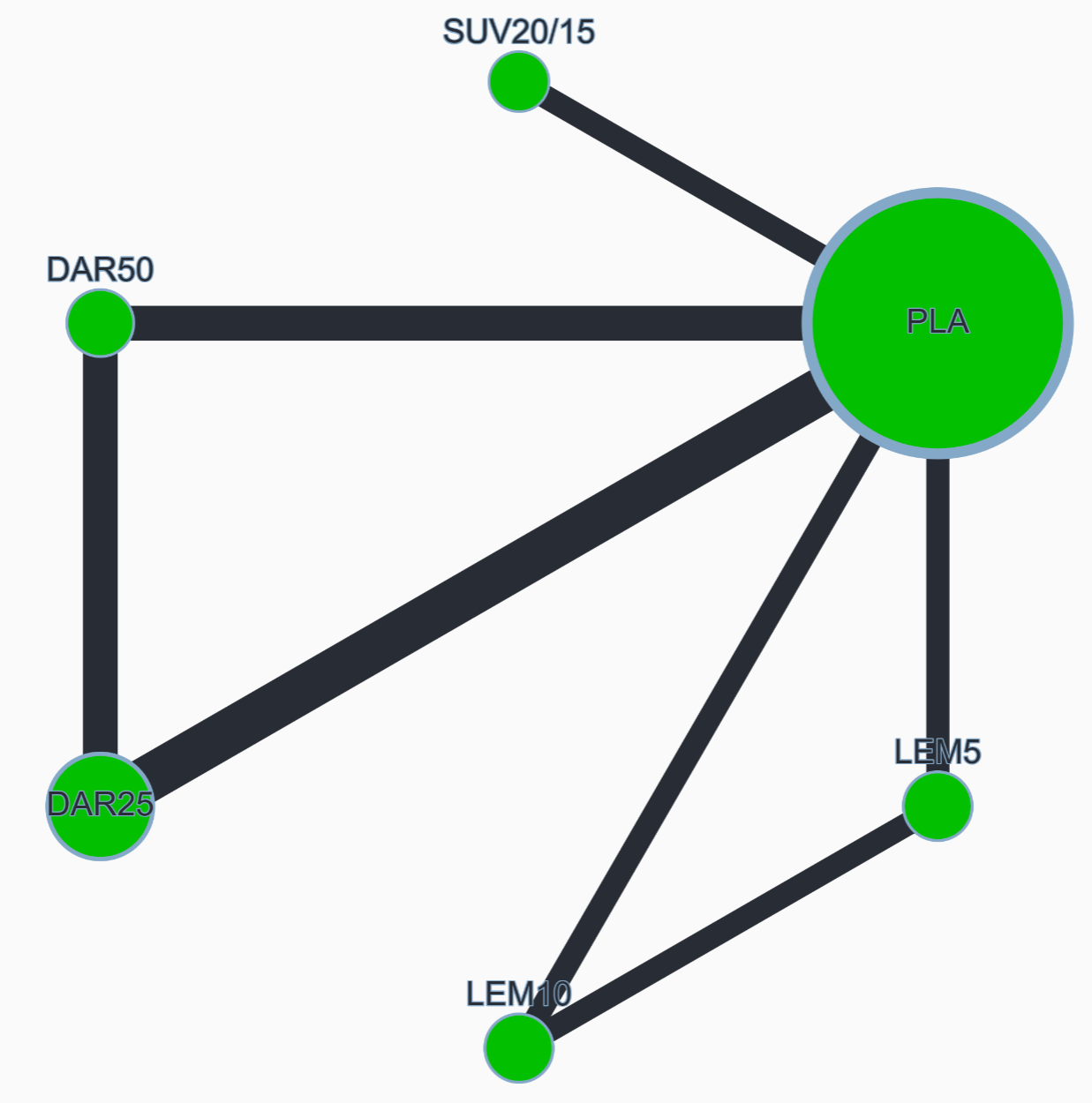
**

**League table (SMD with 95% CI)**

| DAR25 | **0.199 (0.082, 0.316)** | -0.070 (-0.228, 0.088) | 0.152 (-0.008, 0.310) | 0.071 (-0.082, 0.224) | **-0.276 (-0.374, -0.178)** |
| --- | --- | --- | --- | --- | --- |
|  | DAR50 | **-0.269 (-0.440, -0.098)** | -0.047 (-0.219, 0.125) | -0.128 (-0.295, 0.038) | **-0.475 (-0.593, -0.357)** |
|  |  | LEM5 | **0.221 (0.100, 0.342)** | 0.141 (-0.030, 0.312) | **-0.206 (-0.330, -0.082)** |
|  |  |  | LEM10 | -0.081 (-0.253, 0.091) | **-0.428 (-0.553, -0.302)** |
|  |  |  |  | SUV20/15 | **-0.347 (-0.464, -0.229)** |
|  |  |  |  |  | PLA |

**Global heterogeneity**

Between study variance (𝜏^2^): 0.000 (heterogeneity assessment: low)

**Random-effects design-by-treatment interaction model**

χ^2^ statistic: 0.610 (1 degrees of freedom), P value: 0.435

**Local heterogeneity (I^2^) and incoherence (SIDE test)**

|  | NMA SMD | Direct SMD | I^2^ | Indirect SMD | P value (SIDE test) |
| --- | --- | --- | --- | --- | --- |
| DAR25 vs DAR50 | 0.199 (0.082, 0.316) | 0.213 (0.091, 0.336) | 0.0% | 0.049 (-0.346, 0.443) | 0.435 |
| DAR25 vs PLA |  | -0.276 (-0.374, -0.178) | 0.0% |  |  |
| DAR50 vs PLA | -0.475 (-0.593, -0.357) | -0.461 (-0.584, -0.337) | 0.0% | -0.616 (-1.002, -0.229) | 0.454 |
| LEM5 vs LEM10 |  | 0.222 (0.101, 0.343) | 0.0% |  |  |
| LEM5 vs PLA | -0.206 (-0.330, -0.082) | -0.209 (-0.334, -0.085) | **56.6%** | 1.329 (-1.328, 3.987) | 0.257 |
| LEM10 vs PLA | -0.428 (-0.553, -0.302) | -0.429 (-0.555, -0.304) | **68.1%** | 0.566 (-2.357, 3.489) | 0.505 |
| SUV20/15 vs PLA |  | -0.347 (-0.465, -0.229) | 0.0% |  |  |

**Funnel plot (all drugs vs. placebo)**


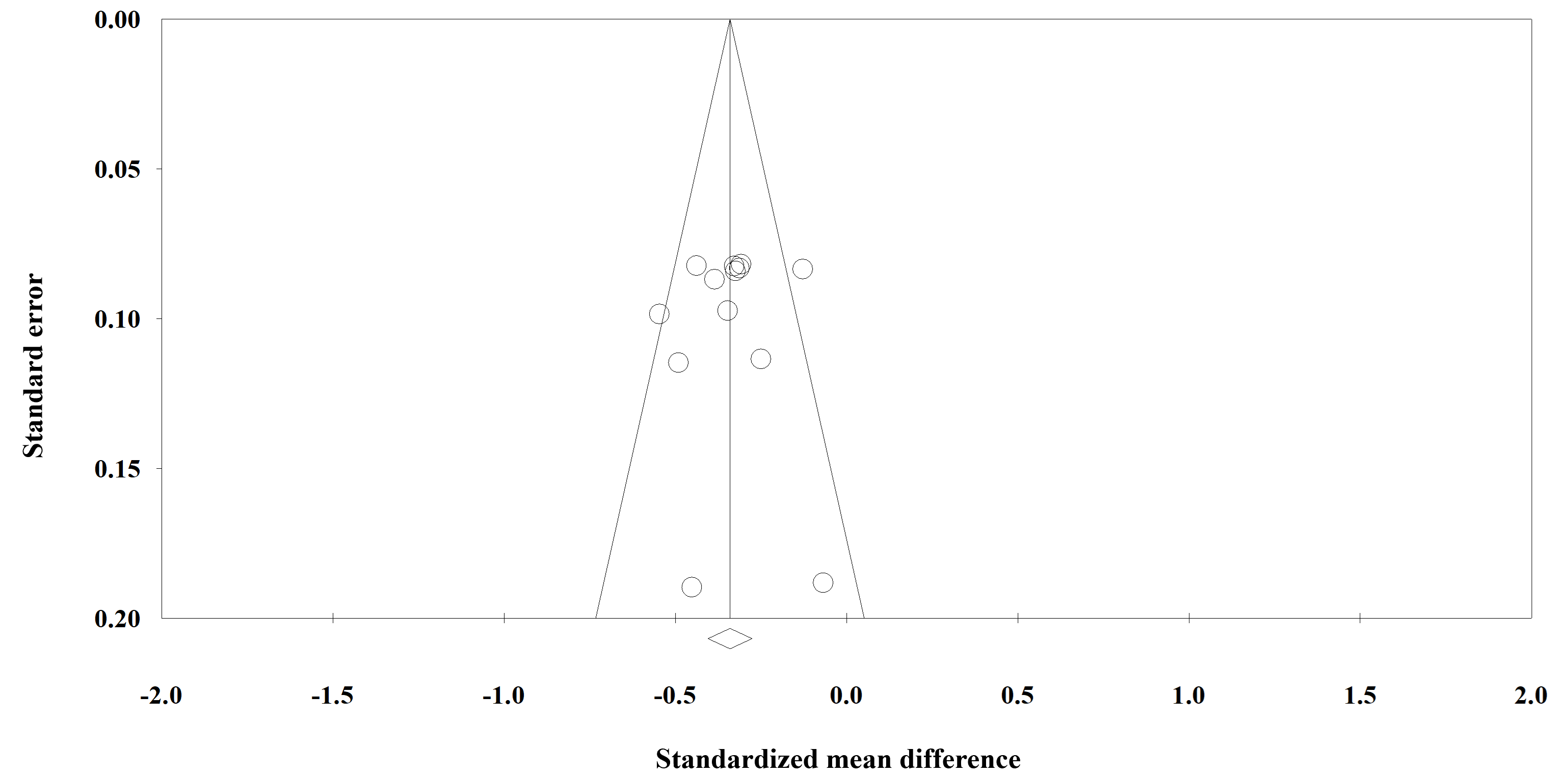


**Meta-regression analysis (the placebo was the control)**

| Potential moderators | β (95% CrI) |
| --- | --- |
| Mean age (k = 8) | -43.976 (-130.521, 88.715) |
| Proportion of females (k = 8) | -13.250 (-180.005, 157.304) |
| Proportion of elderly patients (k = 8) | 17.653 (-32.977, 95.740) |
| Publication year (k = 8) | 24.607 (-60.785, 146.658) |
| Total participants (k = 8) | 67.319 (-27.884, 209.537) |

**CINeMA confidence rating**

| Comparison | Number of studies | Within-study bias | Reporting bias | Indirectness | Imprecision | Heterogeneity | Incoherence | Confidence rating |
| --- | --- | --- | --- | --- | --- | --- | --- | --- |
| DAR25 vs DAR50 | 3 | No concerns | Some concerns | No concerns | No concerns | No concerns | No concerns | Moderate |
| DAR25 vs PLA | 4 | No concerns | Some concerns | No concerns | No concerns | No concerns | No concerns | Moderate |
| DAR50 vs PLA | 3 | No concerns | Some concerns | No concerns | No concerns | No concerns | No concerns | Moderate |
| LEM5 vs LEM10 | 2 | No concerns | Some concerns | No concerns | No concerns | No concerns | No concerns | Moderate |
| LEM5 vs PLA | 2 | No concerns | Some concerns | No concerns | No concerns | No concerns | No concerns | Moderate |
| LEM10 vs PLA | 2 | No concerns | Some concerns | No concerns | No concerns | No concerns | No concerns | Moderate |
| SUV20/15 vs PLA | 2 | No concerns | Some concerns | No concerns | No concerns | No concerns | No concerns | Moderate |
| DAR25 vs LEM5 | 0 | No concerns | Some concerns | No concerns | Major concerns | No concerns | No concerns | Very low |
| DAR25 vs LEM10 | 0 | No concerns | Some concerns | No concerns | Major concerns | No concerns | No concerns | Very low |
| DAR25 vs SUV20/15 | 0 | No concerns | Some concerns | No concerns | Major concerns | No concerns | No concerns | Very low |
| DAR50 vs LEM5 | 0 | No concerns | Some concerns | No concerns | No concerns | No concerns | No concerns | Low |
| DAR50 vs LEM10 | 0 | No concerns | Some concerns | No concerns | Major concerns | No concerns | No concerns | Very low |
| DAR50 vs SUV20/15 | 0 | No concerns | Some concerns | No concerns | Major concerns | No concerns | No concerns | Very low |
| LEM5 vs SUV20/15 | 0 | No concerns | Some concerns | No concerns | Major concerns | No concerns | No concerns | Very low |
| LEM10 vs SUV20/15 | 0 | No concerns | Some concerns | No concerns | Major concerns | No concerns | No concerns | Very low |

**Appendix S3.** **Subjective wake after sleep onset at month 1**

8 studies, 4934 participants


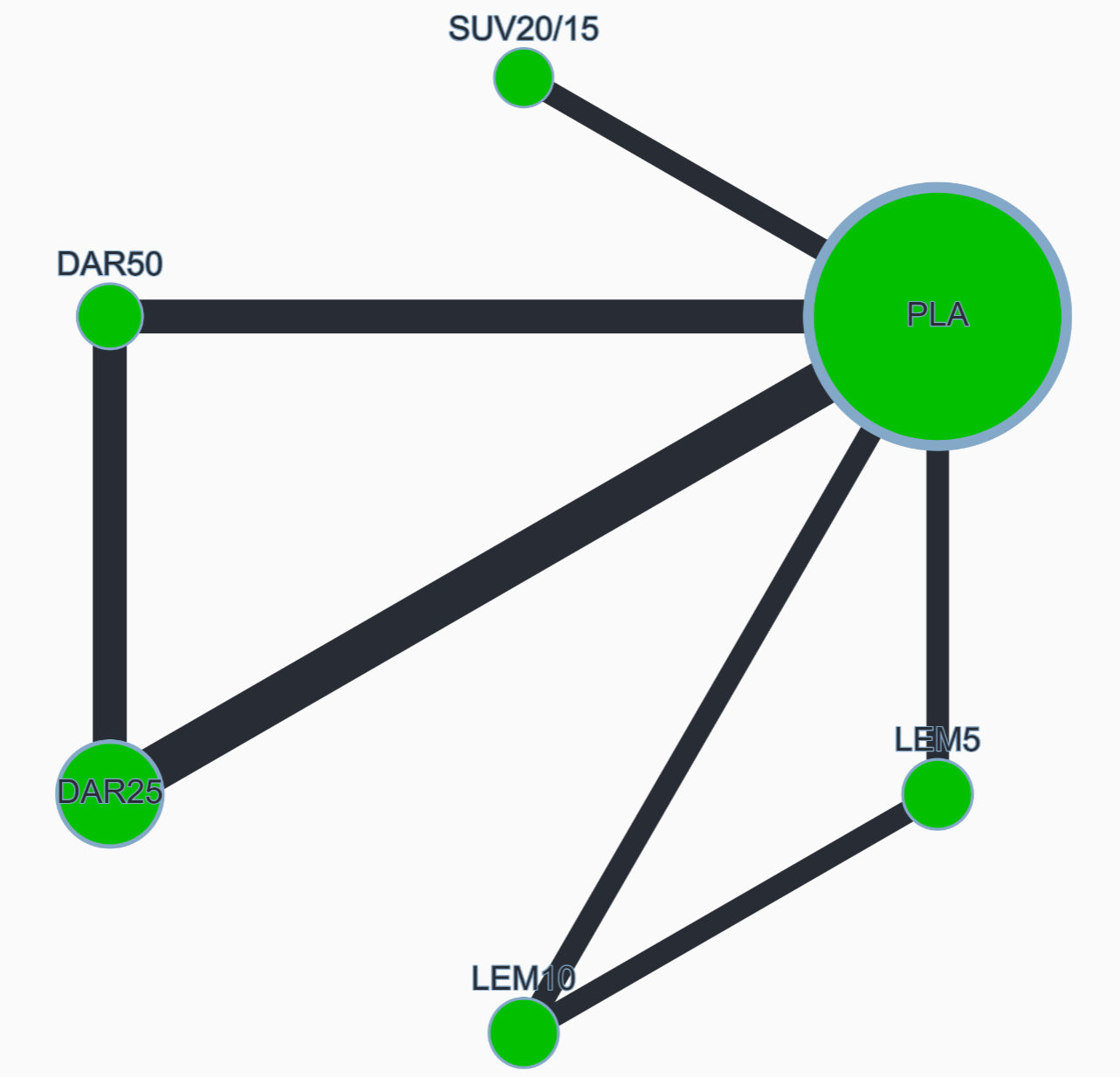


**League table (SMD with 95% CI)**

| DAR25 | 0.023 (-0.093, 0.140) | -0.013 (-0.168, 0.143) | 0.093 (-0.063, 0.249) | 0.056 (-0.096, 0.208) | **-0.138 (-0.234, -0.041)** |
| --- | --- | --- | --- | --- | --- |
|  | DAR50 | -0.036 (-0.205, 0.132) | 0.070 (-0.099, 0.239) | 0.033 (-0.133, 0.198) | **-0.161 (-0.277, -0.045)** |
|  |  | LEM5 | 0.106 (-0.013, 0.224) | 0.069 (-0.100, 0.238) | **-0.125 (-0.247, -0.003)** |
|  |  |  | LEM10 | -0.037 (-0.207, 0.132) | **-0.231 (-0.353, -0.108)** |
|  |  |  |  | SUV20/15 | **-0.194 (-0.311, -0.076)** |
|  |  |  |  |  | PLA |

**Global heterogeneity**

Between study variance (𝜏^2^): 0.000 (heterogeneity assessment: low)

**Random-effects design-by-treatment interaction model**

χ^2^ statistic: 2.633 (1 degrees of freedom), P value: 0.105

**Local heterogeneity (I^2^) and incoherence (SIDE test)**

|  | NMA SMD | Direct SMD | I^2^ | Indirect SMD | P value (SIDE test) |
| --- | --- | --- | --- | --- | --- |
| DAR25 vs DAR50 | 0.023 (-0.093, 0.140) | -0.007 (-0.129, 0.115) | 0.0% | 0.323 (-0.059, 0.704) | 0.106 |
| DAR25 vs PLA |  | -0.138 (-0.234, -0.041) | 0.0% |  |  |
| DAR50 vs PLA | -0.161 (-0.277, -0.045) | -0.192 (-0.314, -0.070) | 0.0% | 0.139 (-0.241, 0.518) | 0.104 |
| LEM5 vs LEM10 |  | 0.106 (-0.013, 0.225) | **61.1%** |  |  |
| LEM5 vs PLA | -0.125 (-0.247, -0.003) | -0.129 (-0.251, -0.007) | 0.0% | 2.827 (-0.293, 5.947) | **0.064** |
| LEM10 vs PLA | -0.231 (-0.353, -0.108) | -0.229 (-0.351, -0.106) | **60.6%** | -1.816(-5.082,1.450) | 0.341 |
| SUV20/15 vs PLA |  | -0.194 (-0.311, -0.076) | 0.0% |  |  |

**CINeMA confidence rating**

| Comparison | Number of studies | Within-study bias | Reporting bias | Indirectness | Imprecision | Heterogeneity | Incoherence | Confidence rating |
| --- | --- | --- | --- | --- | --- | --- | --- | --- |
| DAR25 vs DAR50 | 3 | No concerns | Some concerns | No concerns | Major concerns | No concerns | No concerns | Low |
| DAR25 vs PLA | 4 | No concerns | Some concerns | No concerns | No concerns | No concerns | No concerns | Moderate |
| DAR50 vs PLA | 3 | No concerns | Some concerns | No concerns | No concerns | No concerns | No concerns | Moderate |
| LEM5 vs LEM10 | 2 | No concerns | Some concerns | No concerns | Major concerns | No concerns | No concerns | Low |
| LEM5 vs PLA | 2 | No concerns | Some concerns | No concerns | No concerns | Major concerns | Major concerns | Very low |
| LEM10 vs PLA | 2 | No concerns | Some concerns | No concerns | No concerns | No concerns | No concerns | Moderate |
| SUV20/15 vs PLA | 2 | No concerns | Some concerns | No concerns | No concerns | No concerns | No concerns | Moderate |
| DAR25 vs LEM5 | 0 | No concerns | Some concerns | No concerns | Major concerns | No concerns | No concerns | Very low |
| DAR25 vs LEM10 | 0 | No concerns | Some concerns | No concerns | Major concerns | No concerns | No concerns | Very low |
| DAR25 vs SUV20/15 | 0 | No concerns | Some concerns | No concerns | Major concerns | No concerns | No concerns | Very low |
| DAR50 vs LEM5 | 0 | No concerns | Some concerns | No concerns | Major concerns | No concerns | No concerns | Very low |
| DAR50 vs LEM10 | 0 | No concerns | Some concerns | No concerns | Major concerns | No concerns | No concerns | Very low |
| DAR50 vs SUV20/15 | 0 | No concerns | Some concerns | No concerns | Major concerns | No concerns | No concerns | Very low |
| LEM5 vs SUV20/15 | 0 | No concerns | Some concerns | No concerns | Major concerns | No concerns | No concerns | Very low |
| LEM10 vs SUV20/15 | 0 | No concerns | Some concerns | No concerns | Major concerns | No concerns | No concerns | Very low |

**Appendix S4.** **Insomnia Severity Index scores at month 1**

8 studies, 4967 participants


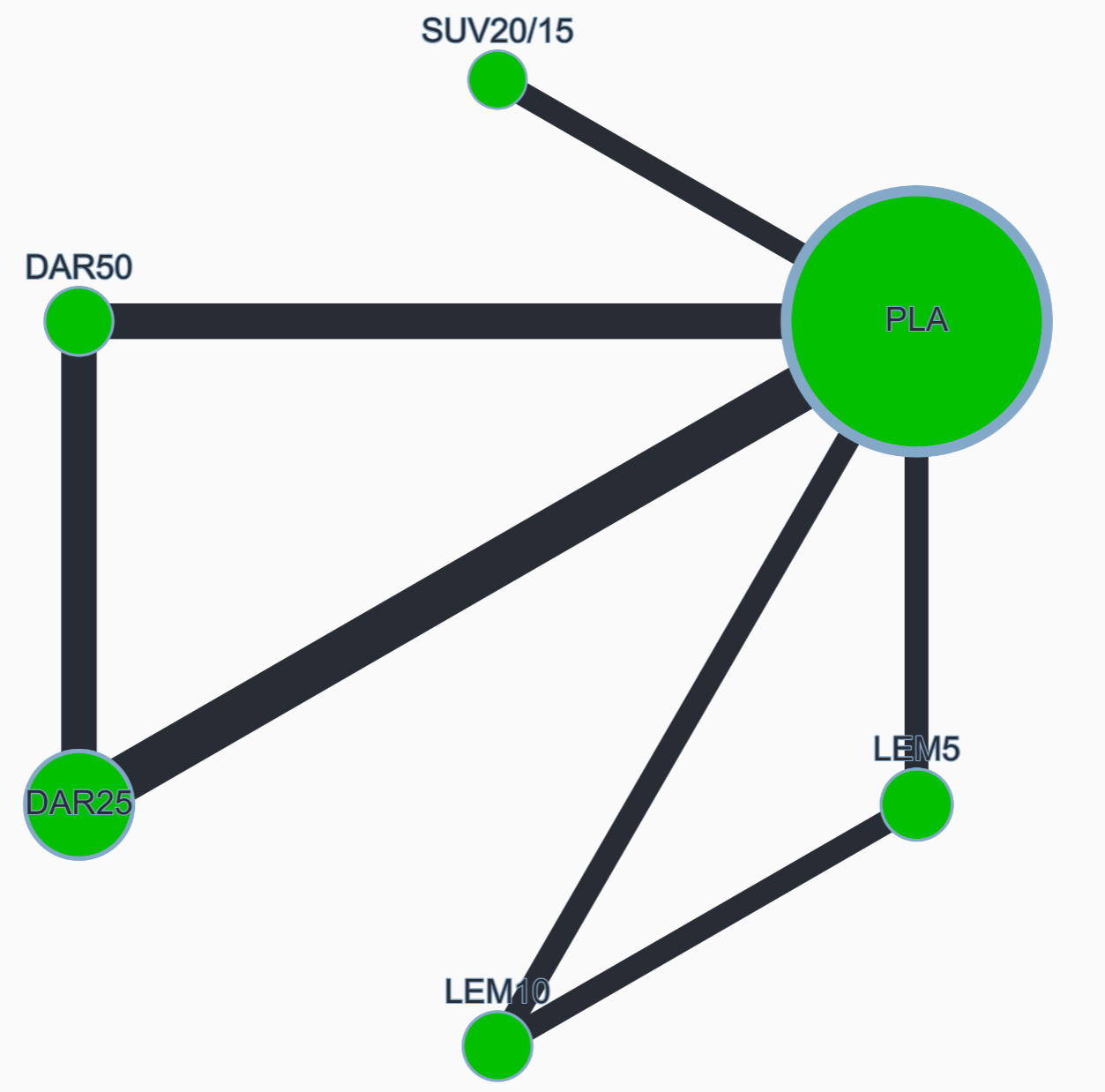


**League table (SMD with 95% CI)**

| DAR25 | 0.070 (-0.045, 0.184) | 0.071 (-0.084, 0.226) | 0.094 (-0.062, 0.250) | 0.100 (-0.053, 0.252) | **-0.212 (-0.308, -0.116)** |
| --- | --- | --- | --- | --- | --- |
|  | DAR50 | 0.002 (-0.166, 0.169) | 0.024 (-0.144, 0.192) | 0.030 (-0.135, 0.195) | **-0.282 (-0.396, -0.167)** |
|  |  | LEM5 | 0.023 (-0.096, 0.141) | 0.029 (-0.141, 0.199) | **-0.283 (-0.405, -0.161)** |
|  |  |  | LEM10 | 0.006 (-0.165, 0.177) | **-0.306 (-0.429, -0.183)** |
|  |  |  |  | SUV20/15 | **-0.312 (-0.430, -0.193)** |
|  |  |  |  |  | PLA |

**Global heterogeneity**

Between study variance (𝜏^2^): 0.000 (heterogeneity assessment: low)

**Random-effects design-by-treatment interaction model**

χ^2^ statistic: 0.341 (1 degrees of freedom), P value: 0.559

**Local heterogeneity (I^2^) and incoherence (SIDE test)**

|  | NMA SMD | Direct SMD | I^2^ | Indirect SMD | P value (SIDE test) |
| --- | --- | --- | --- | --- | --- |
| DAR25 vs DAR50 | 0.070 (-0.045, 0.184) | 0.081 (-0.040, 0.201) | 0.0% | -0.040 (-0.421, 0.341) | 0.554 |
| DAR25 vs PLA |  | -0.212 (-0.308, -0.116) | 0.0% |  |  |
| DAR50 vs PLA | -0.282 (-0.396, -0.167) | -0.271 (-0.391, -0.150) | 0.0% | -0.389 (-0.766, -0.011) | 0.559 |
| LEM5 vs LEM10 |  | 0.023 (-0.096, 0.141) | 0.0% |  |  |
| LEM5 vs PLA | -0.283 (-0.405, -0.161) | -0.283 (-0.405, -0.161) | 0.0% | -0.206 (-3.608, 3.195) | 0.965 |
| LEM10 vs PLA | -0.306 (-0.429, -0.183) | -0.306 (-0.429, -0.183) | 0.0% | -0.034 (-3.003, 2.936) | 0.857 |
| SUV20/15 vs PLA |  | -0.312 (-0.430, -0.193) | 0.0% |  |  |

**CINeMA confidence rating**

| Comparison | Number of studies | Within-study bias | Reporting bias | Indirectness | Imprecision | Heterogeneity | Incoherence | Confidence rating |
| --- | --- | --- | --- | --- | --- | --- | --- | --- |
| DAR25 vs DAR50 | 3 | No concerns | Some concerns | No concerns | Major concerns | No concerns | No concerns | Low |
| DAR25 vs PLA | 4 | No concerns | Some concerns | No concerns | No concerns | No concerns | No concerns | Moderate |
| DAR50 vs PLA | 3 | No concerns | Some concerns | No concerns | No concerns | No concerns | No concerns | Moderate |
| LEM5 vs LEM10 | 2 | No concerns | Some concerns | No concerns | Major concerns | No concerns | No concerns | Low |
| LEM5 vs PLA | 2 | No concerns | Some concerns | No concerns | No concerns | No concerns | No concerns | Moderate |
| LEM10 vs PLA | 2 | No concerns | Some concerns | No concerns | No concerns | No concerns | No concerns | Moderate |
| SUV20/15 vs PLA | 2 | No concerns | Some concerns | No concerns | No concerns | No concerns | No concerns | Moderate |
| DAR25 vs LEM5 | 0 | No concerns | Some concerns | No concerns | Major concerns | No concerns | No concerns | Very low |
| DAR25 vs LEM10 | 0 | No concerns | Some concerns | No concerns | Major concerns | No concerns | No concerns | Very low |
| DAR25 vs SUV20/15 | 0 | No concerns | Some concerns | No concerns | Major concerns | No concerns | No concerns | Very low |
| DAR50 vs LEM5 | 0 | No concerns | Some concerns | No concerns | Major concerns | No concerns | No concerns | Very low |
| DAR50 vs LEM10 | 0 | No concerns | Some concerns | No concerns | Major concerns | No concerns | No concerns | Very low |
| DAR50 vs SUV20/15 | 0 | No concerns | Some concerns | No concerns | Major concerns | No concerns | No concerns | Very low |
| LEM5 vs SUV20/15 | 0 | No concerns | Some concerns | No concerns | Major concerns | No concerns | No concerns | Very low |
| LEM10 vs SUV20/15 | 0 | No concerns | Some concerns | No concerns | Major concerns | No concerns | No concerns | Very low |

**Appendix S5. All-cause discontinuation**

8 studies, 5194 participants


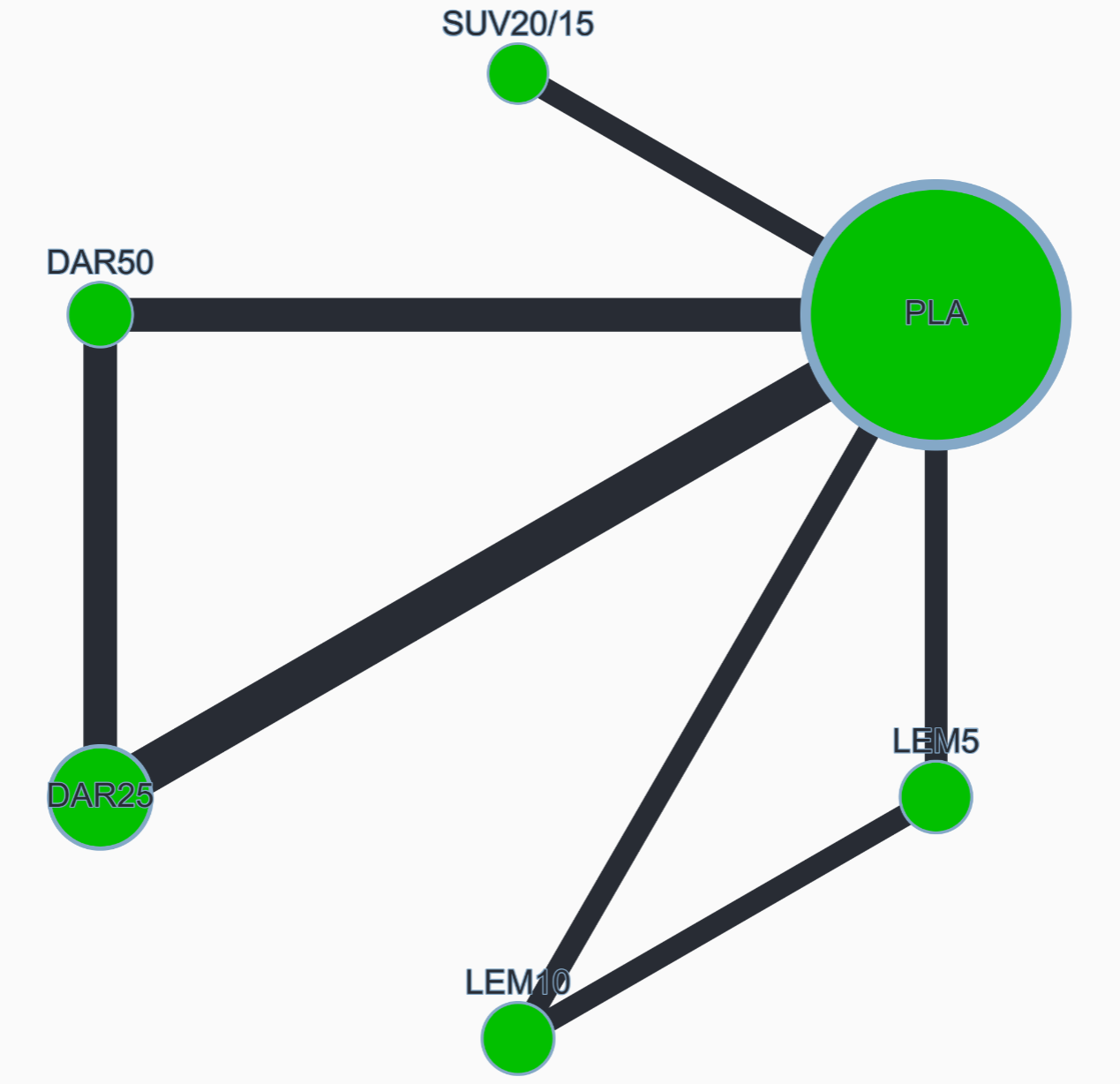


**League table (OR with 95% CI)**

| DAR25 | 1.080 (0.672, 1.733) | 0.961 (0.572, 1.614) | 0.714 (0.429, 1.190) | 1.022 (0.610, 1.710) | 0.963 (0.659, 1.407) |
| --- | --- | --- | --- | --- | --- |
|  | DAR50 | 0.890 (0.497, 1.593) | 0.662 (0.373, 1.175) | 0.946 (0.531, 1.687) | 0.892 (0.562, 1.415) |
|  |  | LEM5 | 0.743 (0.530, 1.043) | 1.063 (0.647, 1.747) | 1.002 (0.703, 1.427) |
|  |  |  | LEM10 | 1.430 (0.878, 2.328) | 1.348 (0.958, 1.895) |
|  |  |  |  | SUV20/15 | 0.942 (0.665, 1.335) |
|  |  |  |  |  | PLA |

**Global heterogeneity**

Between study variance (𝜏^2^): 0.000 (heterogeneity assessment: low)

**Random-effects design-by-treatment interaction model**

χ^2^ statistic: 0.872 (1 degrees of freedom), P value: 0.350

**Sensitivity analysis excluding Kärppä 2020 study**

| DAR25 | 1.080 (0.672, 1.733) | 1.560 (0.562, 4.331) | 1.397 (0.517, 3.778) | 1.022 (0.610, 1.710) | 0.963 (0.659, 1.407) |
| --- | --- | --- | --- | --- | --- |
|  | DAR50 | 1.445 (0.503, 4.148) | 1.294 (0.463, 3.622) | 0.946 (0.531, 1.687) | 0.892 (0.562, 1.415) |
|  |  | LEM5 | 0.896 (0.340, 2.358) | 0.655 (0.239, 1.798) | 0.617 (0.239, 1.592) |
|  |  |  | LEM10 | 0.731 (0.274, 1.954) | 0.689 (0.275, 1.727) |
|  |  |  |  | SUV20/15 | 0.942 (0.665, 1.335) |
|  |  |  |  |  | PLA |

**Global heterogeneity**

Between study variance (𝜏^2^): 0.000 (heterogeneity assessment: low)

**Random-effects design-by-treatment interaction model**

χ^2^ statistic: 0.872 (1 degrees of freedom), P value: 0.350

**Local heterogeneity (I^2^) and incoherence (SIDE test)**

|  | NMA OR | Direct OR | I^2^ | Indirect OR | P value (SIDE test) |
| --- | --- | --- | --- | --- | --- |
| DAR25 vs DAR50 | 1.080 (0.672, 1.733) | 1.000 (0.607, 1.649) | 0.0% | 2.107 (0.479, 9.267) | 0.350 |
| DAR25 vs PLA |  | 0.963 (0.659, 1.407) | 0.0% |  |  |
| DAR50 vs PLA | 0.892 (0.562, 1.415) | 0.836 (0.516, 1.353) | 0.0% | 1.856 (0.367, 9.374) | 0.355 |
| LEM5 vs LEM10 |  | 0.744 (0.531, 1.045) | 0.0% |  |  |
| LEM5 vs PLA | 1.002 (0.703, 1.427) | 1.007 (0.707, 1.436) | 16.3% | 0.000 (0.000, 222.005) | 0.243 |
| LEM10 vs PLA | 1.348 (0.958, 1.895) | 1.350 (0.960, 1.900) | **58.1%** | 0.120 (0.000, 12289.494) | 0.681 |
| SUV20/15 vs PLA |  | 0.942 (0.665, 1.335) | 0.0% |  |  |

**CINeMA confidence rating**

| Comparison | Number of studies | Within-study bias | Reporting bias | Indirectness | Imprecision | Heterogeneity | Incoherence | Confidence rating |
| --- | --- | --- | --- | --- | --- | --- | --- | --- |
| DAR25 vs DAR50 | 3 | No concerns | Some concerns | No concerns | Major concerns | No concerns | No concerns | Low |
| DAR25 vs PLA | 4 | No concerns | Some concerns | No concerns | Major concerns | No concerns | No concerns | Low |
| DAR50 vs PLA | 3 | No concerns | Some concerns | No concerns | Major concerns | No concerns | No concerns | Low |
| LEM5 vs LEM10 | 2 | No concerns | Some concerns | No concerns | Major concerns | No concerns | No concerns | Low |
| LEM5 vs PLA | 2 | No concerns | Some concerns | No concerns | Major concerns | No concerns | No concerns | Low |
| LEM10 vs PLA | 2 | No concerns | Some concerns | No concerns | Major concerns | No concerns | No concerns | Low |
| SUV20/15 vs PLA | 2 | No concerns | Some concerns | No concerns | Major concerns | No concerns | No concerns | Low |
| DAR25 vs LEM5 | 0 | No concerns | Some concerns | No concerns | Major concerns | No concerns | No concerns | Very low |
| DAR25 vs LEM10 | 0 | No concerns | Some concerns | No concerns | Major concerns | No concerns | No concerns | Very low |
| DAR25 vs SUV20/15 | 0 | No concerns | Some concerns | No concerns | Major concerns | No concerns | No concerns | Very low |
| DAR50 vs LEM5 | 0 | No concerns | Some concerns | No concerns | Major concerns | No concerns | No concerns | Very low |
| DAR50 vs LEM10 | 0 | No concerns | Some concerns | No concerns | Major concerns | No concerns | No concerns | Very low |
| DAR50 vs SUV20/15 | 0 | No concerns | Some concerns | No concerns | Major concerns | No concerns | No concerns | Very low |
| LEM5 vs SUV20/15 | 0 | No concerns | Some concerns | No concerns | Major concerns | No concerns | No concerns | Very low |
| LEM10 vs SUV20/15 | 0 | No concerns | Some concerns | No concerns | Major concerns | No concerns | No concerns | Very low |

**Appendix S6. Discontinuation due to adverse events**

8 studies, 5194 participants


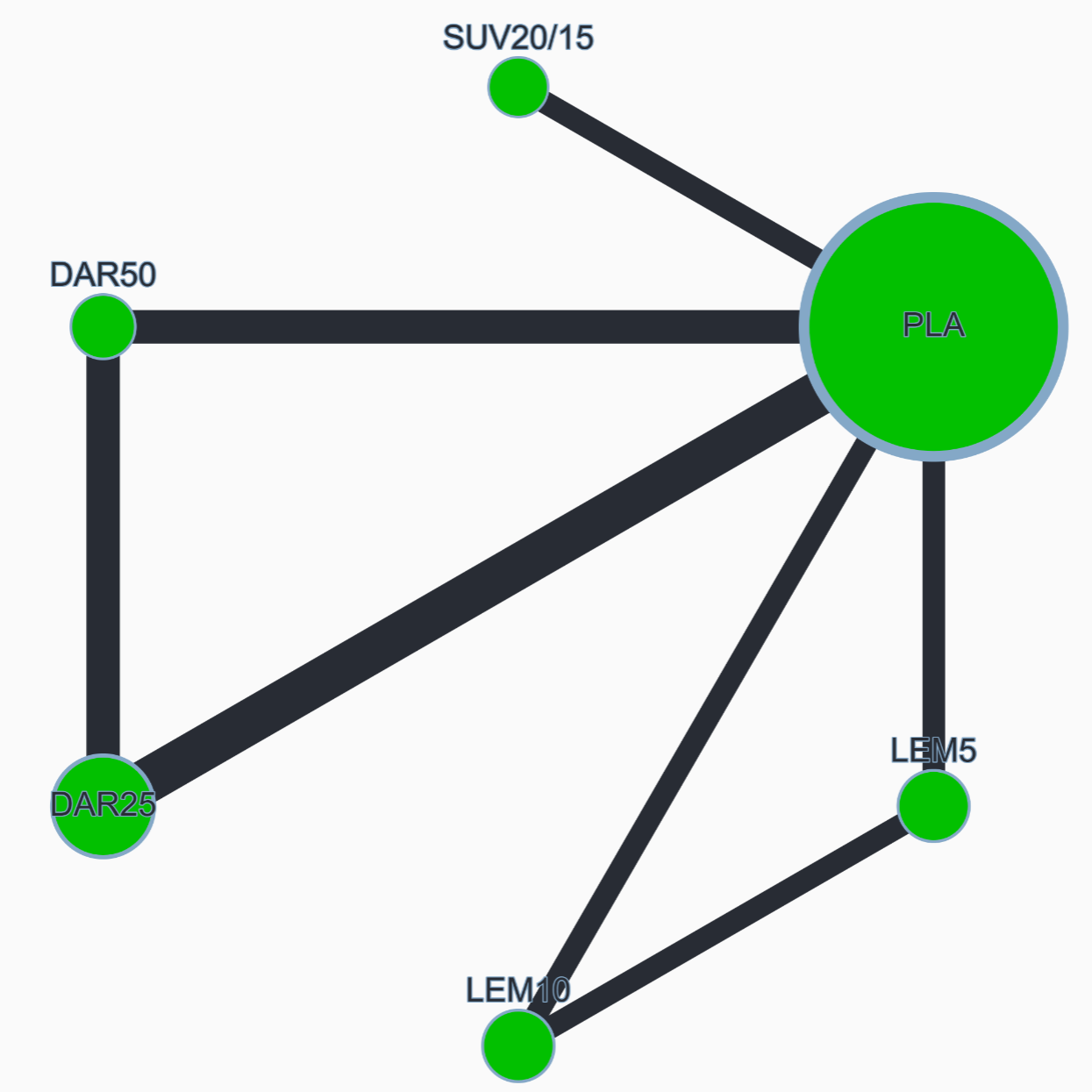


**League table (OR with 95% CI)**

| DAR25 | 0.969 (0.352, 2.665) | 0.613 (0.199, 1.889) | 0.348 (0.121, 1.004) | 0.958 (0.375, 2.448) | 0.639 (0.312, 1.312) |
| --- | --- | --- | --- | --- | --- |
|  | DAR50 | 0.632 (0.174, 2.303) | 0.359 (0.104, 1.235) | 0.988 (0.318, 3.069) | 0.660 (0.253, 1.722) |
|  |  | LEM5 | 0.568 (0.267, 1.208) | 1.563 (0.544, 4.492) | 1.043 (0.439, 2.481) |
|  |  |  | LEM10 | **2.751 (1.028, 7.363)** | 1.837 (0.844, 3.998) |
|  |  |  |  | SUV20/15 | 0.668 (0.365, 1.221) |
|  |  |  |  |  | PLA |

**Global heterogeneity**

Between study variance (𝜏^2^): 0.100 (heterogeneity assessment: moderate to high)

**Random-effects design-by-treatment interaction model**

χ^2^ statistic: 0.059 (1 degrees of freedom), P value: 0.807

**Sensitivity analysis excluding Kärppä 2020 study**

| DAR25 | 0.969 (0.352, 2.665) | 0.815 (0.100, 6.629) | 0.548 (0.079, 3.799) | 0.958 (0.375, 2.448) | 0.639 (0.312, 1.312) |
| --- | --- | --- | --- | --- | --- |
|  | DAR50 | 0.841 (0.094, 7.516) | 0.565 (0.074, 4.339) | 0.988 (0.318, 3.069) | 0.660 (0.253, 1.722) |
|  |  | LEM5 | 0.672 (0.111, 4.053) | 1.175 (0.150, 9.204) | 0.784 (0.110, 5.614) |
|  |  |  | LEM10 | 1.749 (0.262, 11.655) | 1.167 (0.193, 7.051) |
|  |  |  |  | SUV20/15 | 0.668 (0.365, 1.221) |
|  |  |  |  |  | PLA |

**Global heterogeneity**

Between study variance (𝜏^2^): 0.000 (heterogeneity assessment: low)

**Random-effects design-by-treatment interaction model**

χ^2^ statistic: 0.049 (1 degrees of freedom), P value: 0.825

**Local heterogeneity (I^2^) and incoherence (SIDE test)**

|  | NMA OR | Direct OR | I^2^ | Indirect OR | P value (SIDE test) |
| --- | --- | --- | --- | --- | --- |
| DAR25 vs DAR50 | 0.969 (0.352, 2.665) | 1.187 (0.396, 3.559) | 28.9% | 0.311 (0.023, 4.193) | 0.353 |
| DAR25 vs PLA | 0.639 (0.312, 1.312) | 0.636 (0.310, 1.305) | 0.0% | 18.606 (0.000, >1000) | 0.704 |
| DAR50 vs PLA | 0.660 (0.253, 1.722) | 0.612 (0.225, 1.666) | 36.7% | 1.509 (0.055, 41.388) | 0.609 |
| LEM5 vs LEM10 |  | 0.570 (0.268, 1.212) | 0.0% |  |  |
| LEM5 vs PLA |  | 1.046 (0.440, 2.489) | 0.0% |  |  |
| LEM10 vs PLA | 1.837 (0.844, 3.998) | 1.837 (0.844, 4.000) | 0.0% | 1.394 (0.000, >1000) | 0.986 |
| SUV20/15 vs PLA |  | 0.668 (0.365, 1.221) | 41.6% |  |  |

**CINeMA confidence rating**

| Comparison | Number of studies | Within-study bias | Reporting bias | Indirectness | Imprecision | Heterogeneity | Incoherence | Confidence rating |
| --- | --- | --- | --- | --- | --- | --- | --- | --- |
| DAR25 vs DAR50 | 3 | No concerns | Some concerns | No concerns | Major concerns | No concerns | No concerns | Low |
| DAR25 vs PLA | 4 | No concerns | Some concerns | No concerns | Major concerns | No concerns | No concerns | Low |
| DAR50 vs PLA | 3 | No concerns | Some concerns | No concerns | Major concerns | No concerns | No concerns | Low |
| LEM5 vs LEM10 | 2 | No concerns | Some concerns | No concerns | Major concerns | No concerns | No concerns | Low |
| LEM5 vs PLA | 2 | No concerns | Some concerns | No concerns | Major concerns | No concerns | No concerns | Low |
| LEM10 vs PLA | 2 | No concerns | Some concerns | No concerns | Major concerns | No concerns | No concerns | Low |
| SUV20/15 vs PLA | 2 | No concerns | Some concerns | No concerns | Major concerns | No concerns | No concerns | Low |
| DAR25 vs LEM5 | 0 | No concerns | Some concerns | No concerns | Major concerns | No concerns | No concerns | Very low |
| DAR25 vs LEM10 | 0 | No concerns | Some concerns | No concerns | Major concerns | No concerns | No concerns | Very low |
| DAR25 vs SUV20/15 | 0 | No concerns | Some concerns | No concerns | Major concerns | No concerns | No concerns | Very low |
| DAR50 vs LEM5 | 0 | No concerns | Some concerns | No concerns | Major concerns | No concerns | No concerns | Very low |
| DAR50 vs LEM10 | 0 | No concerns | Some concerns | No concerns | Major concerns | No concerns | No concerns | Very low |
| DAR50 vs SUV20/15 | 0 | No concerns | Some concerns | No concerns | Major concerns | No concerns | No concerns | Very low |
| LEM5 vs SUV20/15 | 0 | No concerns | Some concerns | No concerns | Major concerns | No concerns | No concerns | Very low |
| LEM10 vs SUV20/15 | 0 | No concerns | Some concerns | No concerns | No concerns | Major concerns | No concerns | Very low |

**Appendix S7. At least one adverse event**

8 studies, 5161 participants


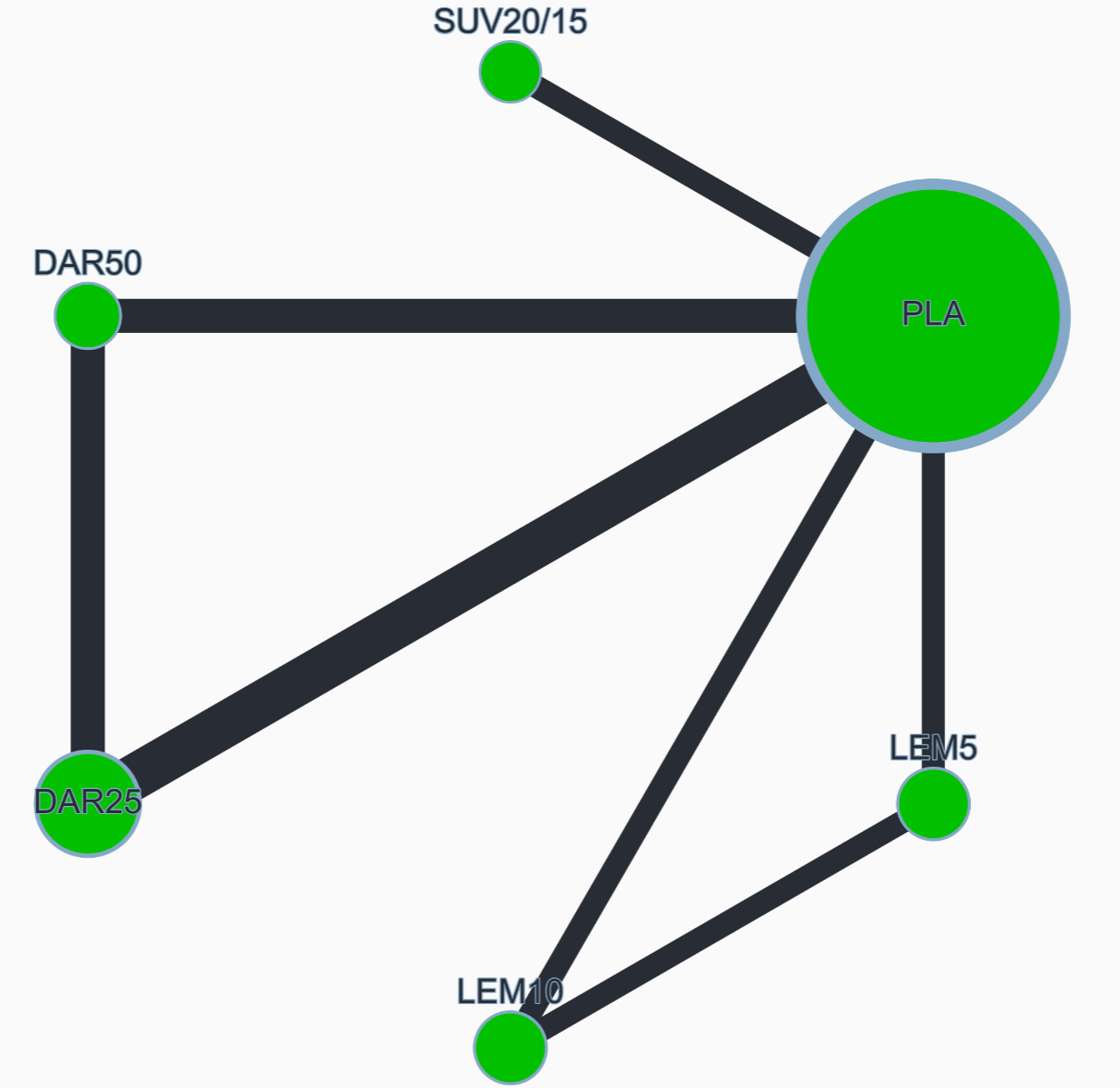


**League table (OR with 95% CI)**

| DAR25 | 1.003 (0.783, 1.286) | 1.176 (0.848, 1.629) | 1.153 (0.833, 1.595) | 1.190 (0.876, 1.618) | 1.175 (0.956, 1.444) |
| --- | --- | --- | --- | --- | --- |
|  | DAR50 | 1.172 (0.822, 1.670) | 1.149 (0.807, 1.636) | 1.186 (0.847, 1.661) | 1.171 (0.914, 1.501) |
|  |  | LEM5 | 0.981 (0.769, 1.250) | 1.012 (0.721, 1.422) | 1.000 (0.776, 1.287) |
|  |  |  | LEM10 | 1.032 (0.736, 1.448) | 1.019 (0.793, 1.310) |
|  |  |  |  | SUV20/15 | 0.987 (0.787, 1.239) |
|  |  |  |  |  | PLA |

**Global heterogeneity**

Between study variance (𝜏^2^): 0.000 (heterogeneity assessment: low)

**Random-effects design-by-treatment interaction model**

χ^2^ statistic: 0.915 (1 degrees of freedom), P value: 0.339

**Sensitivity analysis excluding Kärppä 2020 study**

| DAR25 | 1.003 (0.783, 1.286) | 1.036 (0.654, 1.640) | 0.905 (0.574, 1.427) | 1.190 (0.876, 1.618) | 1.175 (0.956, 1.444) |
| --- | --- | --- | --- | --- | --- |
|  | DAR50 | 1.032 (0.639, 1.669) | 0.903 (0.561, 1.452) | 1.186 (0.847, 1.661) | 1.171 (0.914, 1.501) |
|  |  | LEM5 | 0.874 (0.602, 1.270) | 1.149 (0.718, 1.838) | 1.134 (0.752, 1.711) |
|  |  |  | LEM10 | 1.314 (0.826, 2.093) | 1.298 (0.865, 1.947) |
|  |  |  |  | SUV20/15 | 0.987 (0.787, 1.239) |
|  |  |  |  |  | PLA |

**Global heterogeneity**

Between study variance (𝜏^2^): 0.000 (heterogeneity assessment: low)

**Random-effects design-by-treatment interaction model**

χ^2^ statistic: 0.915 (1 degrees of freedom), P value: 0.339

**Local heterogeneity (I^2^) and incoherence (SIDE test)**

|  | NMA OR | Direct OR | I^2^ | Indirect OR | P value (SIDE test) |
| --- | --- | --- | --- | --- | --- |
| DAR25 vs DAR50 | 1.003 (0.783, 1.286) | 0.960 (0.739, 1.247) | 0.0% | 1.492 (0.678, 3.284) | 0.299 |
| DAR25 vs PLA | 1.175 (0.956, 1.444) | 1.174 (0.956, 1.443) | 16.5% | 2.620 (0.001, >1000) | 0.853 |
| DAR50 vs PLA | 1.171 (0.914, 1.501) | 1.127 (0.868, 1.464) | 0.0% | 1.658 (0.753, 3.651) | 0.364 |
| LEM5 vs LEM10 |  | 0.982 (0.770, 1.252) | 0.0% |  |  |
| LEM5 vs PLA | 1.000 (0.776, 1.287) | 1.007 (0.782, 1.297) | 0.0% | 0.021 (0.000, 6.815) | 0.190 |
| LEM10 vs PLA | 1.019 (0.793, 1.310) | 1.018 (0.792, 1.309) | **55.0%** | 1.389 (0.009, 210.840) | 0.904 |
| SUV20/15 vs PLA |  | 0.987 (0.787, 1.239) | 0.0% |  |  |

**CINeMA confidence rating**

| Comparison | Number of studies | Within-study bias | Reporting bias | Indirectness | Imprecision | Heterogeneity | Incoherence | Confidence rating |
| --- | --- | --- | --- | --- | --- | --- | --- | --- |
| DAR25 vs DAR50 | 3 | No concerns | Some concerns | No concerns | Major concerns | No concerns | No concerns | Low |
| DAR25 vs PLA | 4 | No concerns | Some concerns | No concerns | Major concerns | No concerns | No concerns | Low |
| DAR50 vs PLA | 3 | No concerns | Some concerns | No concerns | Major concerns | No concerns | No concerns | Low |
| LEM5 vs LEM10 | 2 | No concerns | Some concerns | No concerns | Major concerns | No concerns | No concerns | Low |
| LEM5 vs PLA | 2 | No concerns | Some concerns | No concerns | Major concerns | No concerns | No concerns | Low |
| LEM10 vs PLA | 2 | No concerns | Some concerns | No concerns | Major concerns | No concerns | No concerns | Low |
| SUV20/15 vs PLA | 2 | No concerns | Some concerns | No concerns | Major concerns | No concerns | No concerns | Low |
| DAR25 vs LEM5 | 0 | No concerns | Some concerns | No concerns | Major concerns | No concerns | No concerns | Very low |
| DAR25 vs LEM10 | 0 | No concerns | Some concerns | No concerns | Major concerns | No concerns | No concerns | Very low |
| DAR25 vs SUV20/15 | 0 | No concerns | Some concerns | No concerns | Major concerns | No concerns | No concerns | Very low |
| DAR50 vs LEM5 | 0 | No concerns | Some concerns | No concerns | Major concerns | No concerns | No concerns | Very low |
| DAR50 vs LEM10 | 0 | No concerns | Some concerns | No concerns | Major concerns | No concerns | No concerns | Very low |
| DAR50 vs SUV20/15 | 0 | No concerns | Some concerns | No concerns | Major concerns | No concerns | No concerns | Very low |
| LEM5 vs SUV20/15 | 0 | No concerns | Some concerns | No concerns | Major concerns | No concerns | No concerns | Very low |
| LEM10 vs SUV20/15 | 0 | No concerns | Some concerns | No concerns | Major concerns | No concerns | No concerns | Very low |

**Appendix S8. Somnolence**

8 studies, 5161 participants


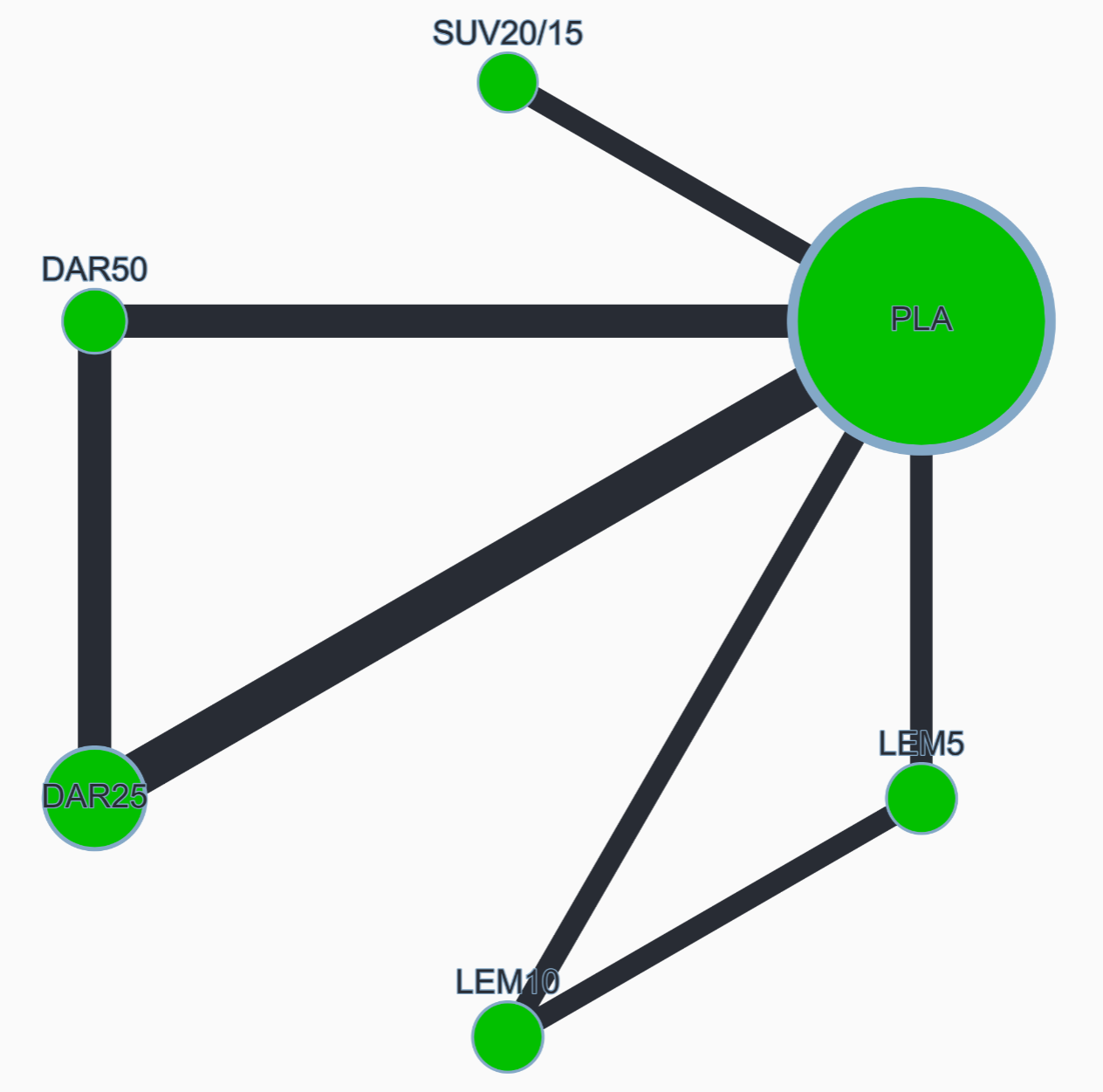


**League table (OR with 95% CI)**

| DAR25 | 1.036 (0.549, 1.957) | 0.498 (0.190, 1.303) | **0.303 (0.118, 0.777)** | 0.914 (0.404, 2.069) | **1.945 (1.052, 3.598)** |
| --- | --- | --- | --- | --- | --- |
|  | DAR50 | 0.480 (0.172, 1.341) | **0.292 (0.107, 0.800)** | 0.882 (0.362, 2.151) | 1.877 (0.922, 3.821) |
|  |  | LEM5 | **0.608 (0.397, 0.932)** | 1.837 (0.736, 4.585) | **3.907 (1.864, 8.191)** |
|  |  |  | LEM10 | **3.019 (1.235, 7.376)** | **6.421 (3.145, 13.109)** |
|  |  |  |  | SUV20/15 | **2.127 (1.243, 3.641)** |
|  |  |  |  |  | PLA |

**Global heterogeneity**

Between study variance (𝜏^2^): 0.040 (heterogeneity assessment: moderate to high)

**Random-effects design-by-treatment interaction model**

χ^2^ statistic: 0.256 (1 degrees of freedom), P value: 0.613

**Sensitivity analysis excluding Kärppä 2020 study**

| DAR25 | 1.040 (0.542, 1.994) | 0.879 (0.230, 3.359) | 0.497 (0.137, 1.796) | 0.915 (0.393, 2.128) | **1.943 (1.037, 3.640)** |
| --- | --- | --- | --- | --- | --- |
|  | DAR50 | 0.845 (0.211, 3.389) | 0.478 (0.126, 1.816) | 0.880 (0.351, 2.205) | 1.868 (0.905, 3.857) |
|  |  | LEM5 | 0.565 (0.254, 1.260) | 1.041 (0.280, 3.868) | 2.211 (0.676, 7.230) |
|  |  |  | LEM10 | 1.842 (0.525, 6.462) | **3.911 (1.274, 12.001)** |
|  |  |  |  | SUV20/15 | **2.123 (1.208, 3.734)** |
|  |  |  |  |  | PLA |

**Global heterogeneity**

Between study variance (𝜏^2^): 0.001 (heterogeneity assessment: low)

**Random-effects design-by-treatment interaction model**

χ^2^ statistic: 0.238 (1 degrees of freedom), P value: 0.626

**Local heterogeneity (I^2^) and incoherence (SIDE test)**

|  | NMA OR | Direct OR | I^2^ | Indirect OR | P value (SIDE test) |
| --- | --- | --- | --- | --- | --- |
| DAR25 vs DAR50 | 1.036 (0.549, 1.957) | 1.039 (0.539, 2.004) | 45.7% | 0.996 (0.080, 12.460) | 0.974 |
| DAR25 vs PLA | 1.945 (1.052, 3.598) | 1.962 (1.060, 3.631) | 0.0% | 0.005 (0.000, >1000) | 0.467 |
| DAR50 vs PLA | 1.877 (0.922, 3.821) | 1.600 (0.745, 3.436) | 33.8% | 5.246 (0.753, 36.551) | 0.265 |
| LEM5 vs LEM10 |  | 0.607 (0.396, 0.930) | 0.0% |  |  |
| LEM5 vs PLA | 3.907 (1.864, 8.191) | 3.945 (1.876, 8.296) | 38.6% | 1.230 (0.000, >1000) | 0.780 |
| LEM10 vs PLA | 6.421 (3.145, 13.109) | 6.481 (3.172, 13.243) | 30.0% | 0.120 (0.000, >1000) | 0.598 |
| SUV20/15 vs PLA |  | 2.127 (1.243, 3.641) | 18.1% |  |  |

**CINeMA confidence rating**

| Comparison | Number of studies | Within-study bias | Reporting bias | Indirectness | Imprecision | Heterogeneity | Incoherence | Confidence rating |
| --- | --- | --- | --- | --- | --- | --- | --- | --- |
| DAR25 vs DAR50 | 3 | No concerns | Some concerns | No concerns | Major concerns | No concerns | No concerns | Low |
| DAR25 vs PLA | 4 | No concerns | Some concerns | No concerns | No concerns | Major concerns | No concerns | Low |
| DAR50 vs PLA | 3 | No concerns | Some concerns | No concerns | Major concerns | No concerns | No concerns | Low |
| LEM5 vs LEM10 | 2 | No concerns | Some concerns | No concerns | No concerns | Major concerns | No concerns | Low |
| LEM5 vs PLA | 2 | No concerns | Some concerns | No concerns | No concerns | No concerns | No concerns | Moderate |
| LEM10 vs PLA | 2 | No concerns | Some concerns | No concerns | No concerns | No concerns | No concerns | Moderate |
| SUV20/15 vs PLA | 2 | No concerns | Some concerns | No concerns | No concerns | No concerns | No concerns | Moderate |
| DAR25 vs LEM5 | 0 | No concerns | Some concerns | No concerns | Major concerns | No concerns | No concerns | Very low |
| DAR25 vs LEM10 | 0 | No concerns | Some concerns | No concerns | No concerns | No concerns | No concerns | Low |
| DAR25 vs SUV20/15 | 0 | No concerns | Some concerns | No concerns | Major concerns | No concerns | No concerns | Very low |
| DAR50 vs LEM5 | 0 | No concerns | Some concerns | No concerns | Major concerns | No concerns | No concerns | Very low |
| DAR50 vs LEM10 | 0 | No concerns | Some concerns | No concerns | No concerns | No concerns | No concerns | Low |
| DAR50 vs SUV20/15 | 0 | No concerns | Some concerns | No concerns | Major concerns | No concerns | No concerns | Very low |
| LEM5 vs SUV20/15 | 0 | No concerns | Some concerns | No concerns | Major concerns | No concerns | No concerns | Very low |
| LEM10 vs SUV20/15 | 0 | No concerns | Some concerns | No concerns | No concerns | No concerns | No concerns | Low |

**Appendix S9. Dizziness**

7 studies, 4214 participants


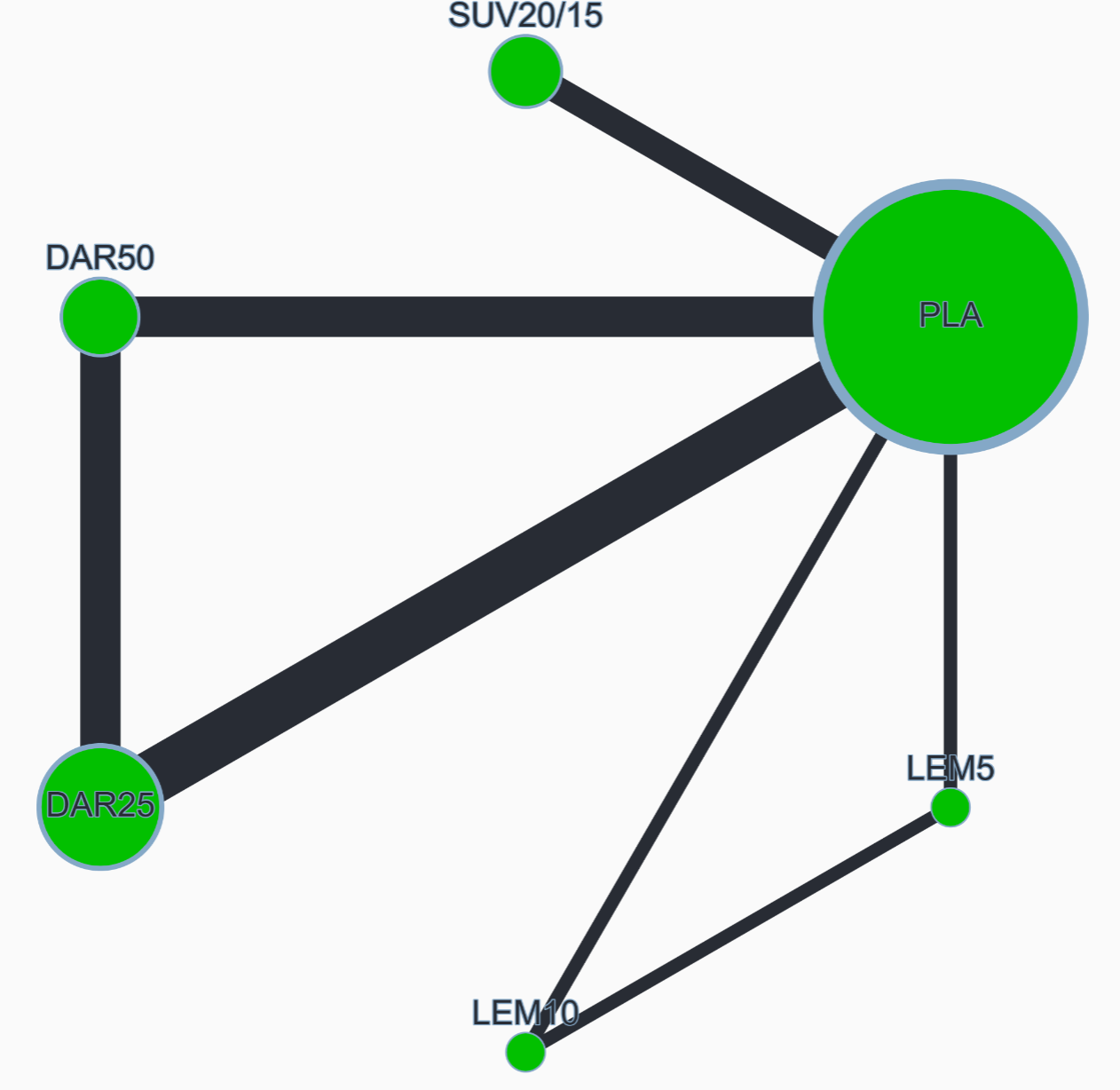


**League table (OR with 95% CI)**

| DAR25 | 0.658 (0.263, 1.649) | 2.603 (0.453, 14.972) | 3.950 (0.577, 27.040) | 1.235 (0.402, 3.792) | 1.522 (0.627, 3.694) |
| --- | --- | --- | --- | --- | --- |
|  | DAR50 | 3.954 (0.651, 24.011) | 5.999 (0.834, 43.155) | 1.875 (0.562, 6.256) | 2.312 (0.859, 6.218) |
|  |  | LEM5 | 1.517 (0.251, 9.153) | 0.474 (0.090, 2.488) | 0.585 (0.129, 2.641) |
|  |  |  | LEM10 | 0.313 (0.050, 1.969) | 0.385 (0.070, 2.124) |
|  |  |  |  | SUV20/15 | 1.233 (0.620, 2.451) |
|  |  |  |  |  | PLA |

**Global heterogeneity**

Between study variance (𝜏^2^): 0.000 (heterogeneity assessment: low)

**Random-effects design-by-treatment interaction model**

χ^2^ statistic: 0.001 (1 degrees of freedom), P value: 0.975

The primary analysis did not include the long-term study (Kärppä 2020).

**Local heterogeneity (I^2^) and incoherence (SIDE test)**

|  | NMA OR | Direct OR | I^2^ | Indirect OR | P value (SIDE test) |
| --- | --- | --- | --- | --- | --- |
| DAR25 vs DAR50 | 0.658 (0.263, 1.649) | 0.676 (0.249, 1.833) | 0.0% | 0.570 (0.054, 5.954) | 0.895 |
| DAR25 vs PLA | 1.522 (0.627, 3.694) | 1.474 (0.591, 3.679) | 0.0% | 2.515 (0.067, 94.273) | 0.779 |
| DAR50 vs PLA | 2.312 (0.859, 6.218) | 2.522 (0.816, 7.791) | 0.0% | 1.728 (0.220, 13.577) | 0.752 |
| LEM5 vs LEM10 |  | 1.517 (0.251, 9.153) | na |  |  |
| LEM5 vs PLA |  | 0.585 (0.129, 2.641) | na |  |  |
| LEM10 vs PLA |  | 0.385 (0.070, 2.124) | na |  |  |
| SUV20/15 vs PLA |  | 1.233 (0.620, 2.451) | 0.0% |  |  |

**CINeMA confidence rating**

| Comparison | Number of studies | Within-study bias | Reporting bias | Indirectness | Imprecision | Heterogeneity | Incoherence | Confidence rating |
| --- | --- | --- | --- | --- | --- | --- | --- | --- |
| DAR25 vs DAR50 | 3 | No concerns | Some concerns | No concerns | Major concerns | No concerns | No concerns | Low |
| DAR25 vs PLA | 4 | No concerns | Some concerns | No concerns | Major concerns | No concerns | No concerns | Low |
| DAR50 vs PLA | 3 | No concerns | Some concerns | No concerns | Major concerns | No concerns | No concerns | Low |
| LEM5 vs LEM10 | 1 | No concerns | Some concerns | No concerns | Major concerns | No concerns | No concerns | Low |
| LEM5 vs PLA | 1 | No concerns | Some concerns | No concerns | Major concerns | No concerns | No concerns | Low |
| LEM10 vs PLA | 1 | No concerns | Some concerns | No concerns | Major concerns | No concerns | No concerns | Low |
| SUV20/15 vs PLA | 2 | No concerns | Some concerns | No concerns | Major concerns | No concerns | No concerns | Low |
| DAR25 vs LEM5 | 0 | No concerns | Some concerns | No concerns | Major concerns | No concerns | No concerns | Very low |
| DAR25 vs LEM10 | 0 | No concerns | Some concerns | No concerns | Major concerns | No concerns | No concerns | Very low |
| DAR25 vs SUV20/15 | 0 | No concerns | Some concerns | No concerns | Major concerns | No concerns | No concerns | Very low |
| DAR50 vs LEM5 | 0 | No concerns | Some concerns | No concerns | Major concerns | No concerns | No concerns | Very low |
| DAR50 vs LEM10 | 0 | No concerns | Some concerns | No concerns | Major concerns | No concerns | No concerns | Very low |
| DAR50 vs SUV20/15 | 0 | No concerns | Some concerns | No concerns | Major concerns | No concerns | No concerns | Very low |
| LEM5 vs SUV20/15 | 0 | No concerns | Some concerns | No concerns | Major concerns | No concerns | No concerns | Very low |
| LEM10 vs SUV20/15 | 0 | No concerns | Some concerns | No concerns | Major concerns | No concerns | No concerns | Very low |

**Appendix S10. Falls**

8 studies, 5161 participants


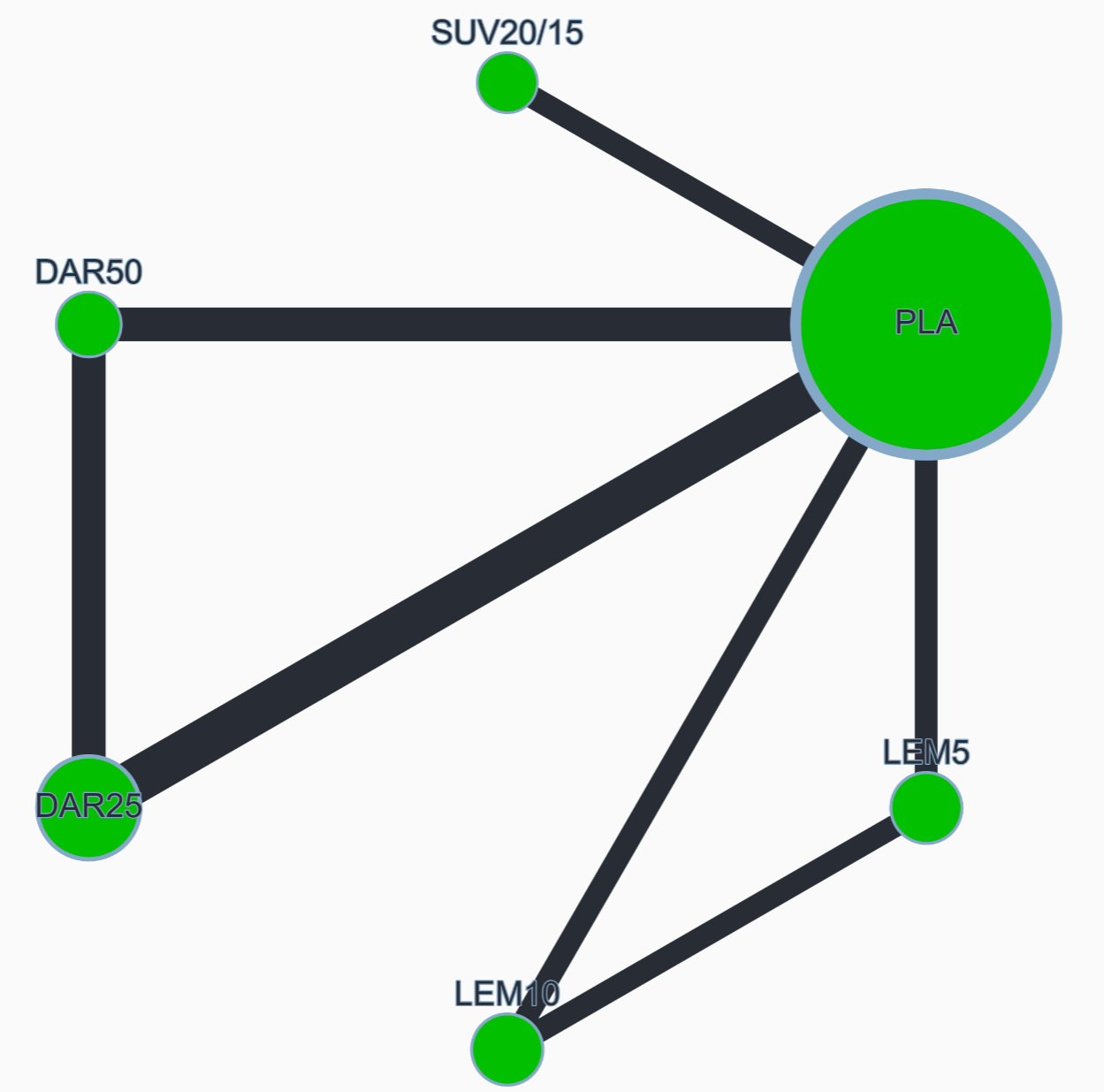


**League table (OR with 95% CI)**

| DAR25 | 0.901 (0.148, 5.495) | 0.472 (0.067, 3.305) | 0.903 (0.121, 6.725) | 0.491 (0.065, 3.733) | 0.455 (0.118, 1.745) |
| --- | --- | --- | --- | --- | --- |
|  | DAR50 | 0.523 (0.059, 4.637) | 1.002 (0.107, 9.376) | 0.545 (0.057, 5.194) | 0.504 (0.095, 2.671) |
|  |  | LEM5 | 1.914 (0.431, 8.509) | 1.041 (0.131, 8.258) | 0.964 (0.236, 3.940) |
|  |  |  | LEM10 | 0.544 (0.065, 4.570) | 0.504 (0.113, 2.237) |
|  |  |  |  | SUV20/15 | 0.926 (0.203, 4.225) |
|  |  |  |  |  | PLA |

**Global heterogeneity**

Between study variance (𝜏^2^): 0.024 (heterogeneity assessment: low to moderate)

**Random-effects design-by-treatment interaction model**

χ^2^ statistic: 1.247 (1 degrees of freedom), P value: 0.264

**Sensitivity analysis excluding Kärppä 2020 study**

| DAR25 | 1.011 (0.193, 5.287) | 0.063 (0.003, 1.463) | 0.581 (0.010, 34.636) | 0.503 (0.091, 2.763) | 0.453 (0.144, 1.424) |
| --- | --- | --- | --- | --- | --- |
|  | DAR50 | 0.062 (0.002, 1.672) | 0.575 (0.009, 38.313) | 0.497 (0.070, 3.521) | 0.449 (0.100, 2.002) |
|  |  | LEM5 | 9.205 (0.493, 171.829) | 7.961 (0.328, 193.002) | 7.183 (0.385, 134.169) |
|  |  |  | LEM10 | 0.865 (0.014, 53.357) | 0.780 (0.015, 39.488) |
|  |  |  |  | SUV20/15 | 0.902 (0.255, 3.190) |
|  |  |  |  |  | PLA |

**Global heterogeneity**

Between study variance (𝜏^2^): 0.027 (heterogeneity assessment: low to moderate)

**Random-effects design-by-treatment interaction model**

χ^2^ statistic: 1.850 (1 degrees of freedom), P value: 0.174

**Local heterogeneity (I^2^) and incoherence (SIDE test)**

|  | NMA OR | Direct OR | I^2^ | Indirect OR | P value (SIDE test) |
| --- | --- | --- | --- | --- | --- |
| DAR25 vs DAR50 | 0.901(0.148,5.495) | 0.690(0.095,5.008) | 0.0% | 3.373(0.041,275.614) | 0.519 |
| DAR25 vs PLA | 0.455(0.118,1.745) | 0.508(0.131,1.972) | 0.0% | 0.000(0.000,17.685) | 0.189 |
| DAR50 vs PLA | 0.504(0.095,2.671) | 0.426(0.073,2.506) | 32.3% | 1.854(0.013,256.801) | 0.583 |
| LEM5 vs LEM10 | 1.914(0.431,8.509) | 1.661(0.367,7.513) | 46.5% | 857.396(0.043,17216865.264) | 0.222 |
| LEM5 vs PLA | 0.964(0.236,3.940) | 0.863(0.207,3.604) | **64.3%** | 37.285(0.010,139469.330) | 0.377 |
| LEM10 vs PLA | 0.504(0.113,2.237) | 0.530(0.119,2.359) | 0.0% | 0.000(0.000,62.866) | **0.087** |
| SUV20/15 vs PLA |  | 0.926(0.203,4.225) | 0.0% |  |  |

**CINeMA confidence rating**

| Comparison | Number of studies | Within-study bias | Reporting bias | Indirectness | Imprecision | Heterogeneity | Incoherence | Confidence rating |
| --- | --- | --- | --- | --- | --- | --- | --- | --- |
| DAR25 vs DAR50 | 3 | No concerns | Some concerns | No concerns | Major concerns | No concerns | No concerns | Low |
| DAR25 vs PLA | 4 | No concerns | Some concerns | No concerns | Major concerns | No concerns | No concerns | Low |
| DAR50 vs PLA | 3 | No concerns | Some concerns | No concerns | Major concerns | No concerns | No concerns | Low |
| LEM5 vs LEM10 | 2 | No concerns | Some concerns | No concerns | Major concerns | No concerns | No concerns | Low |
| LEM5 vs PLA | 2 | No concerns | Some concerns | No concerns | Major concerns | No concerns | No concerns | Low |
| LEM10 vs PLA | 2 | No concerns | Some concerns | No concerns | Major concerns | No concerns | No concerns | Low |
| SUV20/15 vs PLA | 2 | No concerns | Some concerns | No concerns | Major concerns | No concerns | No concerns | Low |
| DAR25 vs LEM5 | 0 | No concerns | Some concerns | No concerns | Major concerns | No concerns | No concerns | Very low |
| DAR25 vs LEM10 | 0 | No concerns | Some concerns | No concerns | Major concerns | No concerns | No concerns | Very low |
| DAR25 vs SUV20/15 | 0 | No concerns | Some concerns | No concerns | Major concerns | No concerns | No concerns | Very low |
| DAR50 vs LEM5 | 0 | No concerns | Some concerns | No concerns | Major concerns | No concerns | No concerns | Very low |
| DAR50 vs LEM10 | 0 | No concerns | Some concerns | No concerns | Major concerns | No concerns | No concerns | Very low |
| DAR50 vs SUV20/15 | 0 | No concerns | Some concerns | No concerns | Major concerns | No concerns | No concerns | Very low |
| LEM5 vs SUV20/15 | 0 | No concerns | Some concerns | No concerns | Major concerns | No concerns | No concerns | Very low |
| LEM10 vs SUV20/15 | 0 | No concerns | Some concerns | No concerns | Major concerns | No concerns | No concerns | Very low |

**Appendix S11. Headache**

7 studies, 4672 participants


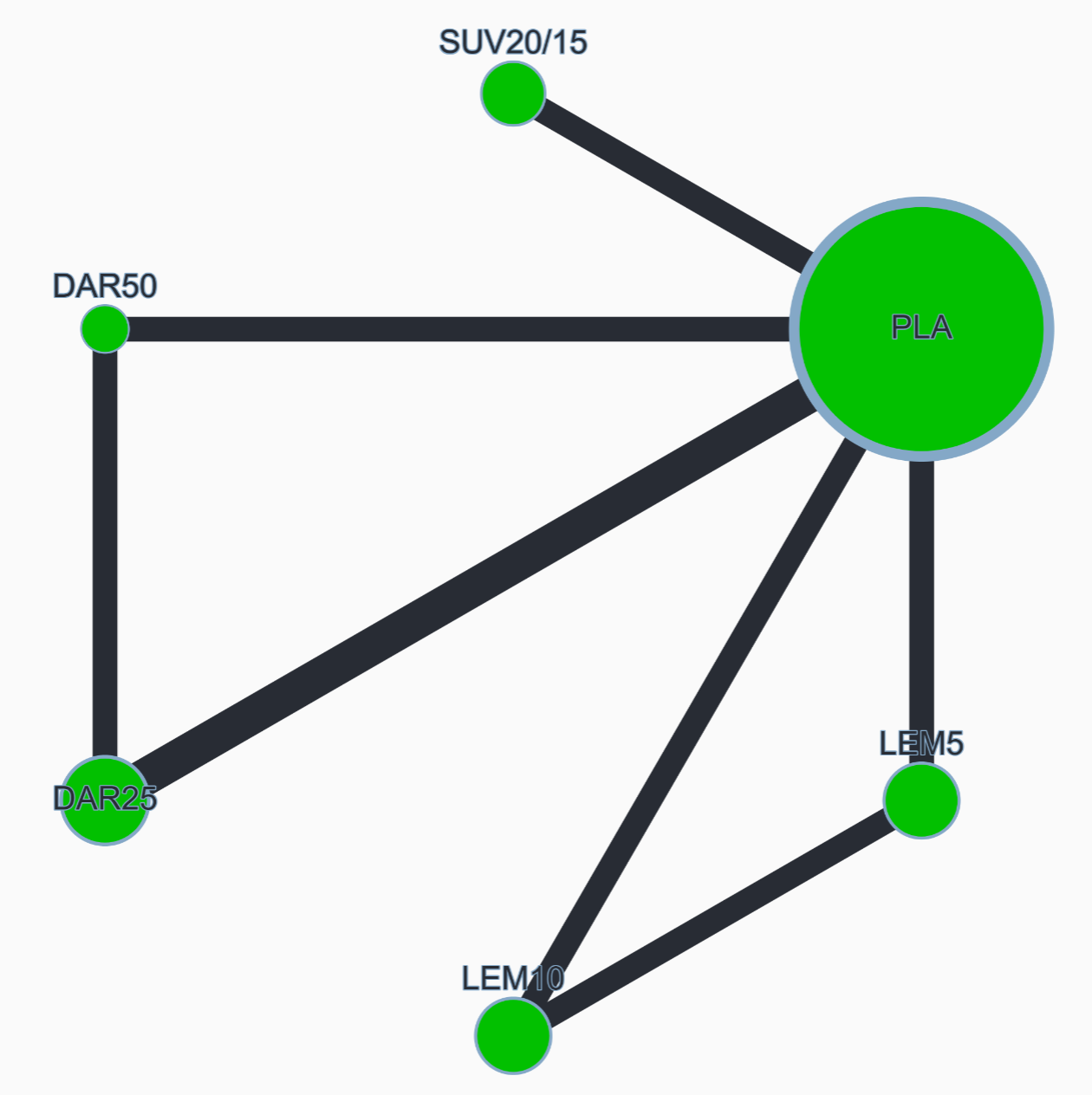


**League table (OR with 95% CI)**

| DAR25 | 0.851 (0.478, 1.514) | 1.190 (0.588, 2.407) | 1.613 (0.782, 3.326) | 1.164 (0.578, 2.342) | 1.473 (0.865, 2.508) |
| --- | --- | --- | --- | --- | --- |
|  | DAR50 | 1.398 (0.640, 3.058) | 1.896 (0.852, 4.219) | 1.368 (0.629, 2.977) | 1.732 (0.921, 3.256) |
|  |  | LEM5 | 1.356 (0.855, 2.151) | 0.978 (0.512, 1.870) | 1.238 (0.780, 1.966) |
|  |  |  | LEM10 | 0.722 (0.370, 1.408) | 0.913 (0.559, 1.492) |
|  |  |  |  | SUV20/15 | 1.266 (0.804, 1.993) |
|  |  |  |  |  | PLA |

**Global heterogeneity**

Between study variance (𝜏^2^): 0.000 (heterogeneity assessment: low)

**Random-effects design-by-treatment interaction model**

χ^2^ statistic: 0.055 (1 degrees of freedom), P value: 0.815

**Sensitivity analysis excluding Kärppä 2020 study**

| DAR25 | 0.851 (0.478, 1.514) | 1.431 (0.573, 3.577) | 1.916 (0.739, 4.971) | 1.164 (0.578, 2.342) | 1.473 (0.865, 2.508) |
| --- | --- | --- | --- | --- | --- |
|  | DAR50 | 1.682 (0.633, 4.470) | 2.253 (0.819, 6.198) | 1.368 (0.629, 2.977) | 1.732 (0.921, 3.256) |
|  |  | LEM5 | 1.339 (0.637, 2.815) | 0.813 (0.340, 1.947) | 1.029 (0.488, 2.170) |
|  |  |  | LEM10 | 0.607 (0.244, 1.512) | 0.769 (0.349, 1.695) |
|  |  |  |  | SUV20/15 | 1.266 (0.804, 1.993) |
|  |  |  |  |  | PLA |

**Global heterogeneity**

Between study variance (𝜏^2^): 0.000 (heterogeneity assessment: low)

**Random-effects design-by-treatment interaction model**

χ^2^ statistic: 0.055 (1 degrees of freedom), P value: 0.815

**Local heterogeneity (I^2^) and incoherence (SIDE test)**

|  | NMA OR | Direct OR | I^2^ | Indirect OR | P value (SIDE test) |
| --- | --- | --- | --- | --- | --- |
| DAR25 vs DAR50 | 0.851 (0.478, 1.514) | 0.866 (0.473, 1.586) | 0.0% | 0.712 (0.107, 4.757) | 0.848 |
| DAR25 vs PLA | 1.473 (0.865, 2.508) | 1.477 (0.865, 2.520) | 0.0% | 1.165 (0.005, 253.205) | 0.931 |
| DAR50 vs PLA | 1.732 (0.921, 3.256) | 1.838 (0.912, 3.705) | 0.2% | 1.343 (0.315, 5.724) | 0.703 |
| LEM5 vs LEM10 |  | 1.356 (0.855, 2.150) | 0.0% |  |  |
| LEM5 vs PLA |  | 1.238 (0.780, 1.966) | 0.0% |  |  |
| LEM10 vs PLA |  | 0.913 (0.559, 1.492) | 0.0% |  |  |
| SUV20/15 vs PLA |  | 1.266 (0.804, 1.993) | 0.0% |  |  |

**CINeMA confidence rating**

| Comparison | Number of studies | Within-study bias | Reporting bias | Indirectness | Imprecision | Heterogeneity | Incoherence | Confidence rating |
| --- | --- | --- | --- | --- | --- | --- | --- | --- |
| DAR25 vs DAR50 | 2 | No concerns | Some concerns | No concerns | Major concerns | No concerns | No concerns | Low |
| DAR25 vs PLA | 3 | No concerns | Some concerns | No concerns | Major concerns | No concerns | No concerns | Low |
| DAR50 vs PLA | 2 | No concerns | Some concerns | No concerns | Major concerns | No concerns | No concerns | Low |
| LEM5 vs LEM10 | 2 | No concerns | Some concerns | No concerns | Major concerns | No concerns | No concerns | Low |
| LEM5 vs PLA | 2 | No concerns | Some concerns | No concerns | Major concerns | No concerns | No concerns | Low |
| LEM10 vs PLA | 2 | No concerns | Some concerns | No concerns | Major concerns | No concerns | No concerns | Low |
| SUV20/15 vs PLA | 2 | No concerns | Some concerns | No concerns | Major concerns | No concerns | No concerns | Low |
| DAR25 vs LEM5 | 0 | No concerns | Some concerns | No concerns | Major concerns | No concerns | No concerns | Very low |
| DAR25 vs LEM10 | 0 | No concerns | Some concerns | No concerns | Major concerns | No concerns | No concerns | Very low |
| DAR25 vs SUV20/15 | 0 | No concerns | Some concerns | No concerns | Major concerns | No concerns | No concerns | Very low |
| DAR50 vs LEM5 | 0 | No concerns | Some concerns | No concerns | Major concerns | No concerns | No concerns | Very low |
| DAR50 vs LEM10 | 0 | No concerns | Some concerns | No concerns | Major concerns | No concerns | No concerns | Very low |
| DAR50 vs SUV20/15 | 0 | No concerns | Some concerns | No concerns | Major concerns | No concerns | No concerns | Very low |
| LEM5 vs SUV20/15 | 0 | No concerns | Some concerns | No concerns | Major concerns | No concerns | No concerns | Very low |
| LEM10 vs SUV20/15 | 0 | No concerns | Some concerns | No concerns | Major concerns | No concerns | No concerns | Very low |

**Appendix S12.** **Nasopharyngitis**

7 studies, 4672 participants


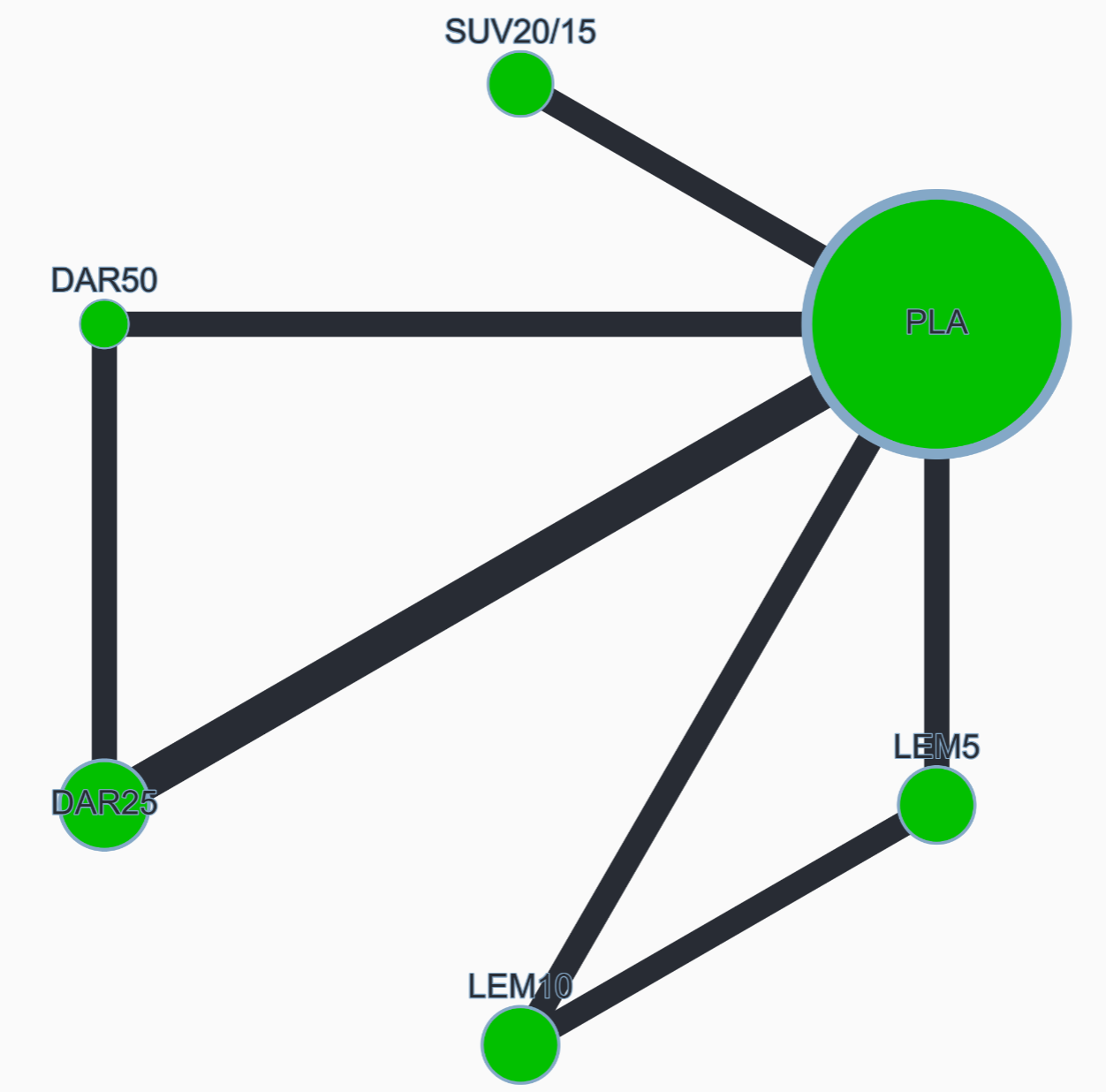


**League table (OR with 95% CI)**

| DAR25 | 0.988 (0.523, 1.868) | 0.995 (0.471, 2.102) | 1.243 (0.576, 2.685) | 1.018 (0.479, 2.159) | 0.862 (0.513, 1.447) |
| --- | --- | --- | --- | --- | --- |
|  | DAR50 | 1.006 (0.443, 2.286) | 1.258 (0.543, 2.915) | 1.029 (0.451, 2.348) | 0.872 (0.470, 1.618) |
|  |  | LEM5 | 1.250 (0.696, 2.245) | 1.023 (0.475, 2.204) | 0.866 (0.505, 1.486) |
|  |  |  | LEM10 | 0.818 (0.372, 1.801) | 0.693 (0.392, 1.225) |
|  |  |  |  | SUV20/15 | 0.847 (0.491, 1.461) |
|  |  |  |  |  | PLA |

**Global heterogeneity**

Between study variance (𝜏^2^): 0.024 (heterogeneity assessment: low to moderate)

**Random-effects design-by-treatment interaction model**

χ^2^ statistic: 0.011 (1 degrees of freedom), P value: 0.917

**Sensitivity analysis excluding Kärppä 2020 study**

| DAR25 | 0.992 (0.556, 1.771) | 0.471 (0.111, 1.999) | 3.401 (0.334, 34.584) | 1.039 (0.523, 2.063) | 0.875 (0.544, 1.405) |
| --- | --- | --- | --- | --- | --- |
|  | DAR50 | 0.475 (0.108, 2.081) | 3.427 (0.330, 35.581) | 1.047 (0.494, 2.222) | 0.881 (0.500, 1.552) |
|  |  | LEM5 | 7.216 (0.882, 59.063) | 2.205 (0.516, 9.418) | 1.856 (0.474, 7.266) |
|  |  |  | LEM10 | 0.306 (0.030, 3.121) | 0.257 (0.027, 2.491) |
|  |  |  |  | SUV20/15 | 0.842 (0.513, 1.382) |
|  |  |  |  |  | PLA |

**Global heterogeneity**

Between study variance (𝜏^2^): 0.000 (heterogeneity assessment: low)

**Random-effects design-by-treatment interaction model**

χ^2^ statistic: 0.094 (1 degrees of freedom), P value: 0.759

**Local heterogeneity (I^2^) and incoherence (SIDE test)**

|  | NMA OR | Direct OR | I^2^ | Indirect OR | P value (SIDE test) |
| --- | --- | --- | --- | --- | --- |
| DAR25 vs DAR50 | 0.988 (0.523, 1.868) | 0.963 (0.485, 1.910) | 9.2% | 1.167 (0.208, 6.536) | 0.839 |
| DAR25 vs PLA | 0.862 (0.513, 1.447) | 0.872 (0.517, 1.469) | 16.2% | 0.398 (0.006, 28.090) | 0.720 |
| DAR50 vs PLA | 0.872 (0.470, 1.618) | 0.905 (0.472, 1.732) | 0.0% | 0.609 (0.081, 4.595) | 0.715 |
| LEM5 vs LEM10 | 1.250 (0.696, 2.245) | 1.207 (0.668, 2.181) | **67.4%** | 6.555 (0.113, 379.214) | 0.419 |
| LEM5 vs PLA | 0.866 (0.505, 1.486) | 0.846 (0.493, 1.452) | 35.5% | 844.631 (0.090, >1000) | 0.140 |
| LEM10 vs PLA | 0.693 (0.392, 1.225) | 0.666 (0.376, 1.180) | 0.0% | 341.620 (0.266,>1000) | **0.089** |
| SUV20/15 vs PLA |  | 0.847 (0.491, 1.461) | 0.0% |  |  |

**CINeMA confidence rating**

| Comparison | Number of studies | Within-study bias | Reporting bias | Indirectness | Imprecision | Heterogeneity | Incoherence | Confidence rating |
| --- | --- | --- | --- | --- | --- | --- | --- | --- |
| DAR25 vs DAR50 | 2 | No concerns | Some concerns | No concerns | Major concerns | No concerns | No concerns | Low |
| DAR25 vs PLA | 3 | No concerns | Some concerns | No concerns | Major concerns | No concerns | No concerns | Low |
| DAR50 vs PLA | 2 | No concerns | Some concerns | No concerns | Major concerns | No concerns | No concerns | Low |
| LEM5 vs LEM10 | 2 | No concerns | Some concerns | No concerns | Major concerns | No concerns | No concerns | Low |
| LEM5 vs PLA | 2 | No concerns | Some concerns | No concerns | Major concerns | No concerns | No concerns | Low |
| LEM10 vs PLA | 2 | No concerns | Some concerns | No concerns | Major concerns | No concerns | No concerns | Low |
| SUV20/15 vs PLA | 2 | No concerns | Some concerns | No concerns | Major concerns | No concerns | No concerns | Low |
| DAR25 vs LEM5 | 0 | No concerns | Some concerns | No concerns | Major concerns | No concerns | No concerns | Very low |
| DAR25 vs LEM10 | 0 | No concerns | Some concerns | No concerns | Major concerns | No concerns | No concerns | Very low |
| DAR25 vs SUV20/15 | 0 | No concerns | Some concerns | No concerns | Major concerns | No concerns | No concerns | Very low |
| DAR50 vs LEM5 | 0 | No concerns | Some concerns | No concerns | Major concerns | No concerns | No concerns | Very low |
| DAR50 vs LEM10 | 0 | No concerns | Some concerns | No concerns | Major concerns | No concerns | No concerns | Very low |
| DAR50 vs SUV20/15 | 0 | No concerns | Some concerns | No concerns | Major concerns | No concerns | No concerns | Very low |
| LEM5 vs SUV20/15 | 0 | No concerns | Some concerns | No concerns | Major concerns | No concerns | No concerns | Very low |
| LEM10 vs SUV20/15 | 0 | No concerns | Some concerns | No concerns | Major concerns | No concerns | No concerns | Very low |

**Appendix S13.** **Upper respiratory tract infection**

7 studies, 4672 participants


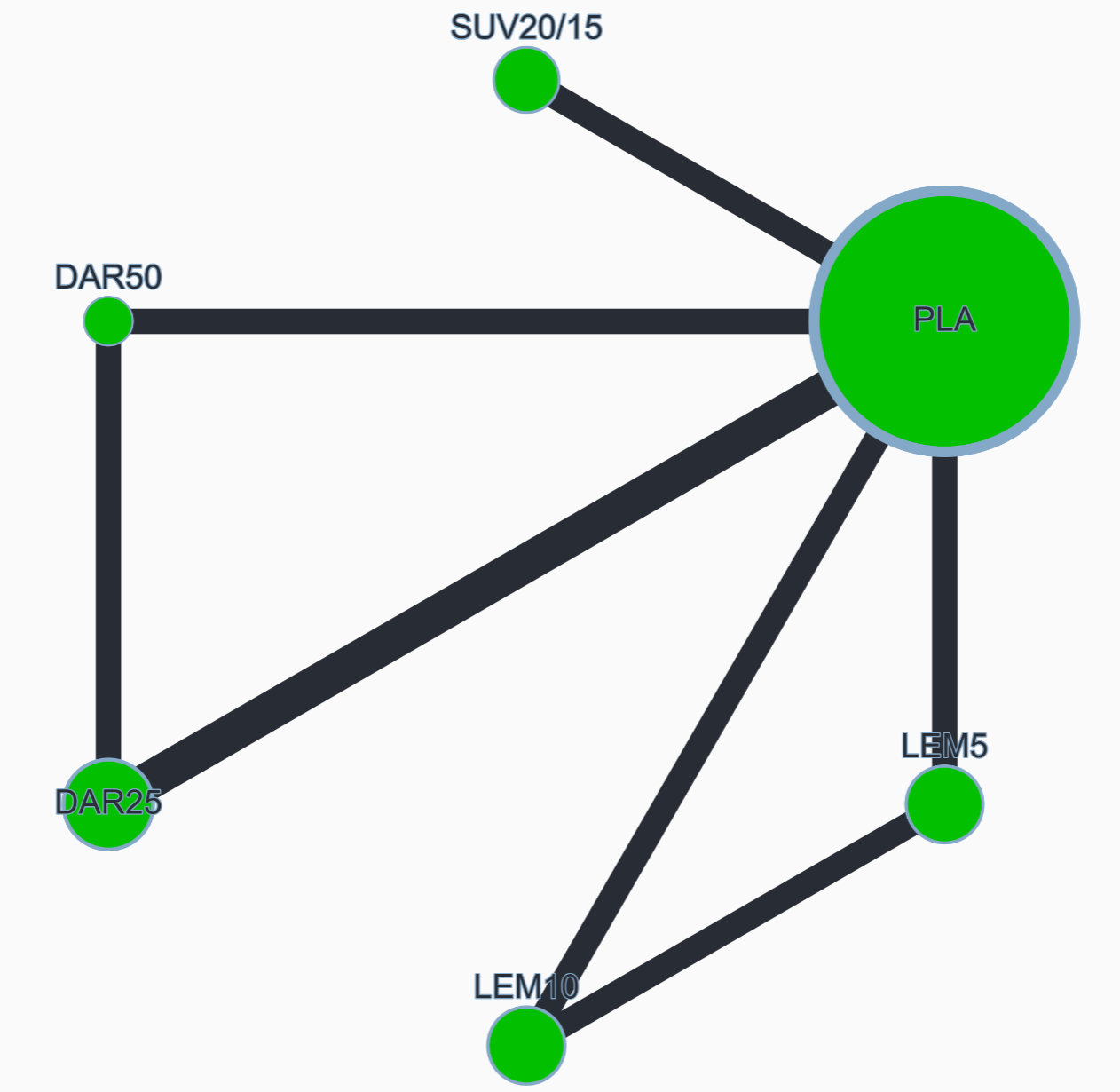


**League table (OR with 95% CI)**

| DAR25 | 1.643 (0.244, 11.064) | 0.435 (0.118, 1.608) | 0.617 (0.158, 2.406) | 0.314 (0.067, 1.473) | 0.563 (0.187, 1.698) |
| --- | --- | --- | --- | --- | --- |
|  | DAR50 | 0.265 (0.037, 1.919) | 0.376 (0.050, 2.821) | 0.191 (0.022, 1.634) | 0.343 (0.054, 2.186) |
|  |  | LEM5 | 1.419 (0.668, 3.015) | 0.723 (0.200, 2.621) | 1.295 (0.642, 2.612) |
|  |  |  | LEM10 | 0.510 (0.133, 1.949) | 0.912 (0.412, 2.023) |
|  |  |  |  | SUV20/15 | 1.791 (0.608, 5.272) |
|  |  |  |  |  | PLA |

**Global heterogeneity**

Between study variance (𝜏^2^): 0.000 (heterogeneity assessment: low)

**Random-effects design-by-treatment interaction model**

χ^2^ statistic: 0.097 (1 degrees of freedom), P value: 0.756

**Sensitivity analysis excluding Kärppä 2020 study**

| DAR25 | 1.643 (0.244, 11.064) | 0.476 (0.088, 2.577) | 2.934 (0.251, 34.350) | 0.314 (0.067, 1.473) | 0.563 (0.187, 1.698) |
| --- | --- | --- | --- | --- | --- |
|  | DAR50 | 0.290 (0.031, 2.752) | 1.786 (0.101, 31.661) | 0.191 (0.022, 1.634) | 0.343 (0.054, 2.186) |
|  |  | LEM5 | 6.161 (0.737, 51.532) | 0.660 (0.124, 3.520) | 1.183 (0.329, 4.247) |
|  |  |  | LEM10 | 0.107 (0.009, 1.242) | 0.192 (0.021, 1.730) |
|  |  |  |  | SUV20/15 | 1.791 (0.608, 5.272) |
|  |  |  |  |  | PLA |

**Global heterogeneity**

Between study variance (𝜏^2^): 0.000 (heterogeneity assessment: low)

**Random-effects design-by-treatment interaction model**

χ^2^ statistic: 0.097 (1 degrees of freedom), P value: 0.756

**Local heterogeneity (I^2^) and incoherence (SIDE test)**

|  | NMA OR | Direct OR | I^2^ | Indirect OR | P value (SIDE test) |
| --- | --- | --- | --- | --- | --- |
| DAR25 vs DAR50 | 1.643 (0.244, 11.064) | 1.614 (0.197, 13.217) | 0.0% | 1.785 (0.019, 165.224) | 0.969 |
| DAR25 vs PLA | 0.563 (0.187, 1.698) | 0.556 (0.182, 1.695) | 0.0% | 1.054 (0.000, 2304.687) | 0.872 |
| DAR50 vs PLA | 0.343 (0.054, 2.186) | 0.436 (0.061, 3.109) | 0.0% | 0.051 (0.000, 13.036) | 0.475 |
| LEM5 vs LEM10 | 1.419 (0.668, 3.015) | 1.472 (0.686, 3.159) | **50.1%** | 0.380 (0.004, 37.989) | 0.569 |
| LEM5 vs PLA |  | 1.287 (0.638, 2.596) | 0.0% |  |  |
| LEM10 vs PLA | 0.912 (0.412, 2.023) | 0.883 (0.393, 1.984) | **53.3%** | 2.349 (0.031, 180.224) | 0.664 |
| SUV20/15 vs PLA |  | 1.791 (0.608, 5.272) | 30.3% |  |  |

**CINeMA confidence rating**

| Comparison | Number of studies | Within-study bias | Reporting bias | Indirectness | Imprecision | Heterogeneity | Incoherence | Confidence rating |
| --- | --- | --- | --- | --- | --- | --- | --- | --- |
| DAR25 vs DAR50 | 2 | No concerns | Some concerns | No concerns | Major concerns | No concerns | No concerns | Low |
| DAR25 vs PLA | 3 | No concerns | Some concerns | No concerns | Major concerns | No concerns | No concerns | Low |
| DAR50 vs PLA | 2 | No concerns | Some concerns | No concerns | Major concerns | No concerns | No concerns | Low |
| LEM5 vs LEM10 | 2 | No concerns | Some concerns | No concerns | Major concerns | No concerns | No concerns | Low |
| LEM5 vs PLA | 2 | No concerns | Some concerns | No concerns | Major concerns | No concerns | No concerns | Low |
| LEM10 vs PLA | 2 | No concerns | Some concerns | No concerns | Major concerns | No concerns | No concerns | Low |
| SUV20/15 vs PLA | 2 | No concerns | Some concerns | No concerns | Major concerns | No concerns | No concerns | Low |
| DAR25 vs LEM5 | 0 | No concerns | Some concerns | No concerns | Major concerns | No concerns | No concerns | Very low |
| DAR25 vs LEM10 | 0 | No concerns | Some concerns | No concerns | Major concerns | No concerns | No concerns | Very low |
| DAR25 vs SUV20/15 | 0 | No concerns | Some concerns | No concerns | Major concerns | No concerns | No concerns | Very low |
| DAR50 vs LEM5 | 0 | No concerns | Some concerns | No concerns | Major concerns | No concerns | No concerns | Very low |
| DAR50 vs LEM10 | 0 | No concerns | Some concerns | No concerns | Major concerns | No concerns | No concerns | Very low |
| DAR50 vs SUV20/15 | 0 | No concerns | Some concerns | No concerns | Major concerns | No concerns | No concerns | Very low |
| LEM5 vs SUV20/15 | 0 | No concerns | Some concerns | No concerns | Major concerns | No concerns | No concerns | Very low |
| LEM10 vs SUV20/15 | 0 | No concerns | Some concerns | No concerns | Major concerns | No concerns | No concerns | Very low |

**Appendix S14. Individual adverse events that were very rare.**

|  | SUV20/15 | LEM5 | LEM10 | DAR25 | DAR50 | Placebo |
| --- | --- | --- | --- | --- | --- | --- |
| Death | 0.000% | 0.000% | 0.000% | 0.119% | 0.000% | 0.047% |
| Suicidal ideation and/or behavior | 0.203% | 0.000% | 0.000% | 0.148% | 0.000% | 0.061% |
| Sleep paralysis | 0.203% | 0.376% | 1.119% | 0.384% | 0.426% | 0.000% |
| Abnormal dreams | 1.826% | 2.229% | 1.274% | 0.119% | 0.000% | 0.675% |
| Nightmare | na | 1.274% | 2.229% | 0.476% | 0.213% | 0.259% |
| Cataplexy | 0.000% | 0.000% | 0.000% | 0.000% | 0.000% | 0.000% |
